# Role of a primary healthcare approach in COVID-19 pandemic response in the eastern Mediterranean region: multicountry case study synthesis

**DOI:** 10.1136/bmjgh-2024-017700

**Published:** 2025-05-30

**Authors:** Fadi El-Jardali, Nour Ataya, Alexandra Edelman, Shehla Zaidi, Robert Marten, Hassan Salah, Hagar Azab, Faraz Khalid, Awad Mataria, Kumanan Rasanathan

**Affiliations:** 1 American University of Beirut, Beirut, Lebanon; 2 Menzies School of Health Research, Alice Springs, Northern Territory, Australia; 3 Global Business School of Health, University College London Faculty of Population Health Sciences, London, UK; 4 The Aga Khan University, Karachi, Pakistan; 5 Alliance for Health Policy and Systems Research, World Health Organization, Geneva, Switzerland; 6 Universal Health Coverage/ Health Systems, World Health Organization Regional Office for the Eastern Mediterranean, Cairo, Egypt; 7 Special Programme on Primary Health Care, World Health Organization, Geneva, Switzerland

**Keywords:** COVID-19, Decision Making, Global Health, Health policies and all other topics

## Abstract

**Introduction:**

Primary healthcare (PHC) can play a critical role in disease preparedness and response. The PHC approach was not always considered in the COVID-19 response in the eastern Mediterranean region (EMR). This article assesses the extent to which a PHC approach was deployed in the pandemic response and identifies barriers, enablers and lessons learnt for strengthening PHC for disease preparedness and response in EMR.

**Methods:**

A multicountry synthesis of 17 case studies from EMR was conducted, using an analytical framework building on the three components of PHC framed by the 2018 Astana Declaration and adapted to enable the analysis of pandemic responses, namely the following. (1) How primary care (PC) and essential public health functions were employed to respond to COVID-19? (2) How multisectoral policy and actions were involved in responding to COVID-19? (3) To what extent was engaging and communicating with communities to leverage community resources effective? Countries were classified into three groups based on the level of socioeconomic development, representing the EMR diversity. Deductive content analysis was conducted.

**Results:**

Findings revealed variations across countries in the application of a PHC approach in pandemic response, with Group 1 countries (higher socioeconomic development) swiftly scaling up PC responses, while Groups 2 and 3 countries prioritised secondary and tertiary care responses. Multisectoral coordination, digital health innovations, cross referrals and expanded disease surveillance commonly emerged as new practices in most EMR countries. Uneven regulatory capacity, inadequately equipped PC workforces and insufficient community engagement were key areas requiring further support.

**Conclusion:**

Priority areas for a comprehensive action agenda for PHC-oriented disease preparedness and response in EMR would benefit from establishing comprehensive PHC-oriented models of care; better resourcing PC; activating an emergency funding pool and strengthening community engagement. Advancing this agenda would contribute to ensuring the health security goal while progressing towards universal health coverage in EMR.

**WHAT IS ALREADY KNOWN ON THIS TOPIC**

- Globally, the COVID-19 pandemic highlighted the critical role that primary healthcare (PHC) can play in managing the unexpected surge of demand on the health system during the pandemic while maintaining access to essential health services.
- A PHC approach, focused on maintaining essential health services, promoting multisectoral collaboration and engaging communities, did not always feature in national pandemic responses in countries across the eastern Mediterranean region (EMR).

**WHAT THIS STUDY ADDS**

- Findings revealed variations across EMR countries with Group 1 countries (higher socioeconomic development) swiftly scaling up primary care (PC) responses, while Groups 2 and 3 countries prioritised secondary and tertiary care responses.
- Multisectoral coordination, digital health innovations, cross referrals and expanded disease surveillance commonly emerged as new practices across most EMR countries.
- Uneven regulatory capacity, inadequately equipped PC workforces and insufficient community engagement are key areas requiring further support.

**HOW THIS STUDY MIGHT AFFECT RESEARCH, PRACTICE OR POLICY**

- We identified three priority areas for a comprehensive agenda for action for PHC-oriented disease preparedness and response in EMR: (1) prioritise a comprehensive strategy to reinforce the role of PHC within health security; (2) improve resourcing of PC systems as a part of preparedness and the acute need for an emergency funding pool for maintaining essential health services and (3) strengthen community engagement as a critical component of integrated community-oriented PC in EMR.
- To do this, we equally identified critical areas for future research: (1) exploring feasibility and acceptability aspects of telemedicine and digital risk communication; (2) identifying and responding to capacity-building needs; (3) creating new entry points for civil society participation and community volunteerism and (4) empowering multistakeholder coordination in terms of key stakeholder motivations, engagement platforms, power-sharing arrangements and legislative and systems support.

## Introduction

 The COVID-19 pandemic posed unprecedented challenges to health systems around the world. In the eastern Mediterranean region (EMR), the first cases of COVID-19 were reported in the United Arab Emirates (UAE) in January 2020.^1^ Since then, more than 23 million confirmed cases of SARS-CoV-2 and 349 545 918 deaths from COVID-19, with an average case fatality rate of 1.5%, were reported, as of February 2023.^2^ There were notable differences in the impact of the pandemic across EMR countries, reflecting variations in country-specific demographics and capacities for detection, diagnostics, reporting and response.^3 4^ The EMR includes 22 countries and territories, ranging from countries affected by protracted conflicts, political unrest, displacement, poverty and fragile health systems to high-income countries.^3 4^ Globally, the COVID-19 pandemic highlighted the critical role that primary healthcare (PHC) can play in managing the unexpected surge of demand during the pandemic while maintaining continued access to essential health services.^5^,^7^ Nonetheless, it remains unclear to what extent the PHC approach featured in the pandemic response in EMR countries, for provision of necessary services and in promoting multisectoral collaboration and community engagement.

In 2015, the Alliance for Health Policy and Systems Research (AHPSR) commissioned a series of PHC systems case studies in 20 low- and middle-income countries (LMICs) globally. Building on these case studies in 2021 and 2022, the Alliance commissioned nearly 50 case studies led by in-country research teams to examine PHC in the context of the COVID-19 pandemic and improve policy efforts to advance the PHC approach in LMICs. These case studies applied the three components of PHC informed by the 2018 Astana Declaration, namely ensuring strong primary care (PC) and essential public health functions (EPHFs) as the core of integrated service delivery, addressing multisectoral policies and actions and empowering people and communities.^8 9^

This article presents findings from a synthesis of 17 case studies from EMR. It aims to (1) assess the extent to which PHC shaped national COVID-19 pandemic responses; (2) explore barriers and enablers to activating PHC to respond to COVID-19 and (3) extract lessons learnt on how to strengthen the role of PHC for future disease preparedness and response in EMR. It provides an agenda for action for EMR policymakers and stakeholders to support national efforts to strengthen PHC, as a central pillar for achieving universal health coverage (UHC) by 2030 while ensuring health security.

## Methods

A multicountry case study synthesis was conducted to summarise evidence on the role of PHC in the COVID-19 response in EMR. The case study synthesis approach involves synthesising a number of individual cases to make the most of the data while making comparisons across cases.^10^,^12^

First, the AHPSR identified in-country research teams through an internal database of existing research partners in countries and supplemented this list in consultation with the WHO Country Offices. While the AHPSR sought to include case studies from all EMR countries, in some countries, they were not able to identify in-country research partners to conduct the case studies. As such, AHPSR commissioned 17 case studies (out of the 22 EMR countries and territories), which formed the basis for this synthesis. Countries included in the synthesis are listed in table 1.

**Table 1:** EMR country groupings* and overall GHSI rankings and COVID-19 epidemiological status

Country groupings*	Overall GHSI ranking†	Total confirmed COVID-19 cases‡	Total deaths‡	CFR‡
Group 1				
KSA	47	829 478	9614	1.2%
Kuwait	59	663 456	2570	0.4%
Oman	73	399 449	4628	1.2%
Qatar	82	494 167	687	0.1%
UAE	56	1 052 029	2349	0.2%
Group 2				
Egypt	87	515 759	24 812	4.8%
I.R. of Iran	97	7 567 906	144 845	1.9%
Iraq	167	2 465 545	25 375	1.0%
Jordan	80	1 746 997	14 122	0.8%
Lebanon	73	1 231 840	10 828	0.9%
Libya	168	507 174	6437	1.3%
Morocco	68	1 272 454	16 296	1.3%
Tunisia	122	1 150 962	29 331	2.5%
Group 3				
Afghanistan	130	209 340	7879	3.8%
Pakistan	105	1 576 998	30 643	1.9%
Sudan	163	63 809	5013	7.9%
Yemen	190	11 945	2159	18.1%

* Countries are grouped based on socioeconomic levels: group 1 countries have the highest level of socioeconomic development, followed by group 2 countries, while group 3 countries have the lowest level of socioeconomic development.^3^

† Overall GHSI rankings are based on GHSI.^30^

‡ Total confirmed COVID-19 cases, total deaths and CFR reported as of February 2023.^2^

CFR, case fatality rate; EMR, eastern Mediterranean region; GHSI, global health security index; I.R. of Iran, Islamic Republic of Iran; KSA, Kingdom of Saudi Arabia; UAE, United Arab Emirates.

In-country research teams employed qualitative research methods to draw on the existing quantitative data, desk review and stakeholder insights on plans, efforts, achievements and challenges of their country’s PHC response to the COVID-19 pandemic from early 2020 to 2022. A key focus of the case studies was on how countries’ PC systems and EPHFs were mobilised in responding to the pandemic and what is needed to strengthen the resilience of the health system for future emergencies. The case studies also examined strategies and processes supporting multisectoral policies and actions and community engagement. In-country research teams reviewed the peer-reviewed and grey literature. Additionally, research teams conducted consultations with government officials, service managers and PHC experts for all country case studies, except Lebanon and Pakistan. In-country research teams triangulated data sources, analysed data thematically and presented findings against the Astana PHC components. Case studies are published on the AHPSR website,^813^,^27^ except for case studies for Libya and Yemen, which are available on request from the authors.

To extract, analyse and synthesise data from the individual case studies, the synthesis researchers used an analytical framework that builds on the 2018 Astana Declaration and the PHC operational framework^7 9 28 29^ (table 2). The analytical framework examines country pandemic responses, enabling factors, barriers and lessons learnt under the three Astana PHC components adapted to enable the analysis of COVID-19 pandemic responses. (1) How PC and EPHFs were employed to respond to COVID-19? (ii) How multisectoral policy and actions were involved in responding to COVID-19? (iii) To what extent engaging and communicating with communities to leverage community resources was effective? We further identified subcomponents from the Astana PHC framework and its accompanying vision document^9 28^ or emerged from the analysis of country case studies (table 2).

**Table 2:** Analytical framework for the role of a PHC approach in pandemic response

Components of a PHC approach*	Subcomponents
How PC and EPHFs were employed to respond to COVID-19?	PC preparedness for COVID-19Policymaking structures leading the COVID-19 responseScaling up and managing services related to COVID-19Maintaining essential PC servicesEPHFs
How multisectoral policy and actions were involved in responding to COVID-19?	Role of PC in multisectoral programmes/initiativesCollaborations involving donors/development partners
To what extent engaging and communicating with communities to leverage community resources was effective?	Role of PC in empowering communitiesCommunity engagement approaches/mechanisms

* PHC approach components are based on the Astana PHC framework^9^ and its accompanying vision document,^28^ adapted to enable the analysis of COVID-19 pandemic responses.

EPHFs, essential public health functions; PC, primary care; PHC, primary healthcare.

For the purpose of facilitating comparisons in the synthesis, we used WHO classification of EMR countries into three groups based on the level of socioeconomic development.^3^ Group 1 countries have the highest level of socioeconomic development, followed by Group 2 countries, while Group 3 countries have the lowest level of socioeconomic development.^3^ Table 1 presents country groupings and rankings on the global health security index (GHSI), as well as COVID-19 epidemiological situation. The GHSI ranks 195 countries across core domains of prevention measures, detection and reporting, response capacity, health system infrastructure and compliance with international norms and financing commitments, and risk mitigation.^30^ Data from the case studies were extracted by a researcher (NA) to an excel spreadsheet based on the components of the analytical framework (table 2). Online supplemental file 1 provides the excel spreadsheet we used to extract data from the case studies, with findings for each case study. The use of a single extraction spreadsheet enabled comparisons within and across country groupings. Two researchers (NA and FE-J) conducted data analysis. We used a deductive content analysis approach to synthesise the data.^4 31^ We categorised findings from the individual case studies into predefined themes that correspond to the main PHC components of the analytical framework (table 2). We further organised the data into specific subcomponents/subthemes that emerged inductively from the data analysis. We then coded data into barriers, facilitators and lessons learnt. This approach enabled a structured analysis, as data were categorised into predefined themes while allowing additional subthemes to emerge outside the framework.^4 31^ The researchers (FE-J, NA and AE) worked together in person and online to discuss and obtain agreement on data analysis and interpretation. This helped to establish clarity on the data analysis process, ensure that relevant data were accounted for, and deepen understanding of the themes.

### Patient and public involvement

This synthesis draws on published case studies. Patients and/or the public were not involved in the design, or conduct, or reporting or dissemination plans of our research.

## Results

Findings are presented below according to the three PHC components adapted to enable the analysis of the COVID-19 pandemic responses (table 2). Findings for each case study are available in online supplemental file 1.

### Component 1: how PC and EPHFs were employed to respond to COVID-19?

#### PC preparedness for the COVID-19 pandemic

Prepandemic EMR countries varied widely in terms of disease preparedness capacity. Group 1 countries, as well as Morocco, Lebanon, Jordan and Egypt, had comparatively higher GHSI ranking at 47–87 positions, followed by I. R. of Iran, Pakistan and Tunisia at 97–122 positions, whereas remaining countries: Afghanistan, Libya, Iraq, Sudan and Yemen at 130–190 positions (table 1). These gaps were further exacerbated with varying policy support and investment in PC structures. In Group 1 countries, there has been strong political commitment and leadership to prioritise PC in recent years (Kingdom of Saudi Arabia (KSA), Kuwait, Oman, Qatar and UAE). In Group 2 countries and in Pakistan, the pandemic occurred in a context of political and economic instability (Iraq, Islamic Republic of Iran (I.R. of Iran) and Lebanon), PC policy fatigue and lack of trust in the public health system (Tunisia) and expansion of the private sector’s role in diagnosis and treatment services (Lebanon, Morocco, Pakistan and Tunisia). There has been insufficient political interest and government leadership to prioritise PC in these countries prior to the pandemic (Iraq, I.R. of Iran, Jordan, Lebanon and Tunisia). For example, in Jordan and Tunisia, while past political commitment had been critical to improving PC and enhancing healthcare access, in recent years, this commitment has waned and the role of PC was limited to prevention. While in Group 3 countries, the COVID-19 outbreak occurred in a context of conflict and reliance on fragmented healthcare systems provided mainly through international support (Afghanistan, Sudan and Yemen). Chronic PC underfunding and shortages in resources (financial, medical and equipment) were major existing challenges in Groups 2 and 3 countries (Afghanistan, Iraq, Jordan, Lebanon, Pakistan, Sudan and Yemen).

At the onset of the pandemic, the majority of countries reported lacking pre-existing mechanisms, structures and national preparedness plans, including PHC targeted or wider health system emergency plans (Afghanistan, I.R. of Iran, Jordan, KSA, Lebanon, Libya, Sudan and Yemen). Among Group 1 countries, Kuwait, Oman, Qatar and the UAE had pre-existing structures and preparedness and response plans that facilitated the role of PC in responding rapidly to the pandemic. However, in Groups 2 and 3 countries, efforts to prepare the health system to respond to the COVID-19 outbreak were supported by international agencies and donor organisations (Jordan, Lebanon, Libya and Sudan) and focused on secondary and tertiary care (Iraq, Jordan, Lebanon, Pakistan, Sudan and Yemen). In yet other cases, emergency taskforces were established, and disease outbreak response and surveillance plans had been developed but weakly implemented due to low prioritisation of funding (Pakistan).

The case studies reported that the pandemic highlighted the importance of a national PHC-targeted preparedness and response plan, with clearly defined roles and responsibilities, lines of authority and financing mechanisms (KSA, Lebanon, Libya, Oman, Pakistan and Sudan), as a key facilitator to engaging PC in responding to emergencies. Resource mobilisation and coordination across stakeholders also emerged as an important function for pandemic response with leadership provided by UN agencies in Group 2 and 3 countries.

#### Policymaking structures leading the COVID-19 response

Some countries swiftly established structures and/or mechanisms to lead the COVID-19 response (Egypt, KSA, Lebanon, Oman, Pakistan and UAE). However, there were notable variations in the extent to which a PHC approach was involved or was represented in national decision-making structures. For example, in Qatar, the Managing Director of PHC centres was assigned to lead the tactical leadership team responsible for ensuring continuity and sustainability of basic services and implementing isolation facilities. While in Lebanon, there was a lack of recognition or explicit role for representatives of PHC centres in national response mechanisms. In Sudan, PHC has been represented in national government-led structures, but PHC service delivery was halted during the pandemic. Countries reported that strong leadership and stewardship functions by the national health authority can harness existing PC resources and capacities to mount an effective emergency response (Lebanon, Pakistan, Qatar and UAE).

#### Scaling up and managing services related to COVID-19

Variations in the extent that a PHC approach was deployed in the COVID-19 response were noted across countries. Early deployment of a PHC approach in the response was reported in most Group 1 countries (Kuwait, Oman, Qatar and UAE).

Segregating care pathways was also reported in Group 1 countries (KSA, Kuwait, Oman, Qatar and UAE) and some Groups 2 and 3 countries (Egypt, I. R. of Iran, Pakistan and Yemen) whereby designated PHC centres screened suspected cases and managed confirmed COVID-19 cases, referred patients to hospital care, followed up COVID-19 cases, conducted contact tracing, health promotion and education and provided COVID-19 vaccinations. In most Groups 2 and 3 countries, priority was allocated to secondary and tertiary care in responding to the pandemic, reflecting historically disproportionate investments in secondary and tertiary care compared with PC (Iraq, I.R. of Iran, Jordan, Lebanon and Pakistan).

The role of a PHC approach in the COVID-19 response was marginalised, although to different degrees (Afghanistan, Egypt, Iraq, Jordan, Lebanon, Pakistan, Sudan, Tunisia and Yemen). For example, in Sudan, the role of PC was restricted to maintaining essential health services, rather than providing COVID-19-related services. In Tunisia, PC workers were assigned to COVID-19 immunisation centres or to mobile teams for home screening. In Pakistan, large community outreach programmes initially struggled to operate in the first wave of COVID-19 and were subsequently equipped with personal protective equipment, training and risk communication messages to respond to subsequent waves. Existing weaknesses in the quality of PHC, shortages in healthcare workers, equipment and medical supplies and lack of preparedness have reportedly contributed to the limited capacity of countries to rapidly scale-up PHC services in response to COVID-19 (Iraq, Lebanon, Morocco, Sudan, Tunisia and Yemen).

Additionally, weak referral systems were reported in Groups 2 and 3 countries (Iraq, I.R. of Iran, Lebanon, Libya and Tunisia). For example, in the I.R. of Iran, patients were able to bypass the voluntary PHC gatekeeping system to access higher levels of care. Countries echoed the need for strong referral systems, with PHC as the first point of contact, supported by an integrated, interoperable health information system to facilitate information sharing across levels of care and between public and private health sectors (Iraq, I.R. of Iran, Jordan and Lebanon).

#### Maintaining essential PC services

Closure of PHC centres (Iraq, Libya, Sudan, Tunisia and Yemen) and disruptions to essential health services (Afghanistan, Iraq, Jordan, Libya, Sudan, Tunisia and Yemen) were reported in Groups 2 and 3 countries, along with the reports of decreased healthcare utilisation particularly for childhood immunisations, pregnancy care visits and management of chronic diseases (Iran, Iraq, Morocco, Oman and Pakistan).

While physical visits continued for essential care in mostly Group 1 countries (Kuwait, Oman, Qatar and UAE) and I.R. of Iran, most countries leveraged telemedicine to maintain essential PHC services (Egypt, I.R. of Iran, KSA, Kuwait, Lebanon, Morocco, Oman, Pakistan, Qatar, Sudan, Tunisia and UAE). Countries identified the scale-up of telemedicine as a key facilitator to addressing the healthcare needs of the population during the pandemic. They emphasised the opportunity to further expand on digital technology as an accessible and cost-effective delivery mechanism to address population needs (Egypt, I.R. of Iran, KSA, Kuwait, Oman, Pakistan, Qatar, Sudan and UAE). Box 1 presents experiences from Oman and Pakistan in leveraging telemedicine during the pandemic.

Box 1 **Leveraging digital technology during the pandemic**

- In Oman**,** Tarassud Plus, a web-based phone application, was developed in collaboration with the Ministry of Telecommunication and the Ministry of Health to provide residents with updated information on COVID-19 status and direct patients to facilities for treatment. The WHO described the application as among the powerful technological solutions deployed in eastern Mediterranean region to track COVID-19 spread and ensure compliance with public health measures. Several challenges were reported in the implementation of this application, including physician dissatisfaction due to the absence of visual cues, inability to perform physical examination and quality of documentation.
- In Pakistan, the COVID-19 pandemic triggered a shift towards digital technology. Telecall helplines supported by virtual triage and telemedicine services were set up and were supported by ambulance services. Public and private providers established telemedicine centres for home-based advice and referrals support. Cell phone tracking systems for contact tracing, risk assessment and referrals were also implemented, with varying levels of uptake among the population due to the fear of quarantine and stigmatisation.

Additionally, countries highlighted the need to integrate mental health and social care services, including geriatric care, youth-friendly services and sexual and reproductive health within the PHC setting to facilitate comprehensive and integrated care (Iraq, Jordan, Lebanon and Pakistan).

#### Essential public health functions

Countries reported that PHC centres provided EPHFs, including risk communication, surveillance, screening, testing and tracing activities in response to the pandemic (Egypt, I.R. of Iran, Kuwait, Lebanon, Libya, Morocco, Oman, Qatar, UAE and Yemen). The roll-out of surveillance and mass testing was largely determined by the availability of laboratories with capacity and provision of digital reporting across facilities, resulting in speedy real-time decision-making (I.R. of Iran, Jordan, Lebanon, Pakistan and Qatar).

A PHC approach was also deployed in awareness raising, health promotion and education about COVID-19 prevention and vaccination (Egypt, I.R. of Iran, KSA, Kuwait, Lebanon, Libya, Morocco, Oman, Qatar and UAE), with digital advocacy and outreach (ie, digital health promotion) used in some countries, such as I.R. of Iran, KSA, Pakistan and UAE, to raise awareness and encourage communities to seek essential healthcare services.

Community-based volunteers in some countries were mobilised to strengthen the PHC approach to responding to COVID-19 (Iraq, I.R. of Iran, Kuwait, Oman, Pakistan and Qatar). For example, in the I.R. of Iran, the PHC network recruited and trained volunteers to support contact tracing, screening, community education and COVID-19 vaccinations. Pakistan’s response drew on high level of volunteerism by the civil society, including philanthropies, NGOs, medical associations, medical students and individual citizens.

Reflecting on lessons learnt, countries called for integrating or strengthening linkages between EPHFs and emergency preparedness and response within PHC systems, including real-time alert systems and strengthening capacity, through staff training and allocation of resources to scale-up response to public health emergencies (Afghanistan, Lebanon, Pakistan, Qatar, Sudan, Tunisia and UAE).

### Component 2: how multisectoral policy and actions were involved in responding to COVID-19?

In Group 1, countries’ multisectoral action, including PC engagement, reportedly helped mobilise national resources in the public and private sectors and across national and local levels to address the pandemic (Kuwait, Oman, Qatar and UAE). For example, in the UAE, existing strategic financial partnerships between public and private sectors, standard operating procedures and memoranda of understanding facilitated coordination between the public and private health sectors to support workforce supply.

However, in Groups 2 and 3 countries, where the transition of governments towards stewardship and engaging with different actors was hampered by political and economic barriers, multisectoral action for COVID-19 response was weaker (Afghanistan, Iraq, Lebanon, Libya and Tunisia) with some exceptions (I.R. of Iran and Pakistan). For example, in Libya, multisectoral coordination at the national level has been inadequate mainly due to the lack of a unified national strategy and political instability. However, at the local level, multisectoral coordination among health offices, municipalities, community members and donors was effective in the provision of essential equipment for PHC facilities. In Pakistan, a high-level multisector national body took carefully coordinated decisions across economic, health, education and public operations sectors. The availability of speedy and reliable digital surveillance data was also seen to facilitate multisectoral decision-making in Pakistan.

Countries from all three groups identified strong leadership (Kuwait, Oman, Pakistan and Qatar), a national PHC strategy or emergency response strategy that emphasises multisectoral action for health (Kuwait, I.R. of Iran, Qatar and UAE) and existing public–private partnerships (Pakistan and UAE) as facilitators to multisectoral action. In Kuwait, for example, multisectoral collaboration built on a pre-existing national emergency plan, which actively engaged PHC and identified clear roles, responsibilities, lines of accountability and chain of command to guide collaboration across PHC and other sectors, such as education and social services, with the aim of reaching vulnerable populations.

Barriers to multisectoral action included weak formal coordination structures lacking clear mandates, roles and responsibilities (Libya), absence of systems for communication and data sharing (Oman) and limited resources and competing interests (Libya and Lebanon). Countries emphasised the need for a coordinated, long-term strategy to sustain multisectoral action (Afghanistan, Iraq, Jordan, Libya and Oman). They also called for integrating a PHC approach as a part of multisectoral action at national and local levels, with clear roles and responsibilities, in times of emergency and non-emergency (Lebanon and Qatar).

### Component 3: to what extent engaging and communicating with communities to leverage community resources was effective?

EMR saw several examples of state-led community engagement with much fewer instances of civil society involvement and volunteerism. Groups 1 and 2 countries provided examples on engaging communities to strengthen the PHC approach to responding to the pandemic (I.R. of Iran, KSA, Kuwait, Oman, Qatar and UAE). In Kuwait and Oman, pre-existing healthy cities, which encourage community engagement for health development, supported PHC centres to deliver medications, raise awareness and organise triage areas and patient flow. In some countries, community-based volunteers, mobile clinics or community health workers within the PHC setting were mobilised to reach vulnerable populations and tackle inequities (Egypt, Iraq, I.R. of Iran, Kuwait, Libya, Morocco, Oman, Pakistan, Qatar and Tunisia). For example, in Qatar, pre-existing channels within PHC centres were used to identify and monitor vulnerable groups in order to engage them and inform surveillance, testing and vaccination policies during the pandemic. Box 2 presents the I.R. of Iran’s community engagement efforts to support a PHC approach to respond to the pandemic.

Box 2 **Community engagement to support a primary healthcare (PHC) approach to responding to the pandemic in I.R. of Iran**

The ‘Each Home, One Health Post’ programme in the I.R. of Iran was launched in PHC centres prior to the COVID-19 pandemic, with the aim to improve health literacy, healthcare access and emergency preparedness and strengthen the health system through public participation. The project assigned one member from each household to receive health education from PHC centres and educate the family on health matters. Health ambassadors currently covering 50% of households were leveraged in the COVID-19 response. Additionally, volunteers were recruited and trained to support PHC centres with contact tracing, screening, community education, home care, referrals and care provision to vulnerable groups.

However, integrated community engagement in PHC delivery and pandemic response was limited. Notable examples include community advisory groups in Lebanon and local community representation in decision-making at the PC level in Port Said in Egypt, which helped identify vulnerable groups and provide bidirectional communication during the pandemic.

In Group 3 countries, despite the availability of community health workers and the pre-existing role of the community in supporting PC in outreach and volunteerism, community engagement in the pandemic response was limited (Afghanistan, Sudan and Yemen). The shifting priorities of funders from providing PHC services to the emergency response (Sudan and Yemen) and the centralised top-down pandemic response limited opportunities for community engagement in the pandemic response in these countries (Sudan).

Pre-established community partnerships that are well prepared for emergencies and relations of trust between PHC centres and the community facilitated community engagement in disease preparedness and response (Egypt, I.R. of Iran, Kuwait, Pakistan and UAE). Key features of effective community engagement were regular and transparent channels of communication with the community (Kuwait, Qatar, UAE and Tunisia). For example, governments in Kuwait and the UAE established long-standing bidirectional strategies for communicating with the community, including the use of telecommunication and media platforms to disseminate information to the public, as well as national surveys and public forums to garner community input for inclusive and participatory decision-making.

Countries highlighted a key opportunity to integrate community engagement as a central component of a PHC-oriented model of care (Egypt, Iraq and Jordan). They reported the need for strengthening coordination among local and international NGOs, UN agencies and national and local policymakers, communities and governmental organisations, within the PHC setting, as a means to reducing fragmentation, increasing responsiveness and promoting social inclusion (Afghanistan, Egypt, I.R. of Iran, Kuwait, Lebanon, Oman and UAE).

## Discussion

This multicountry synthesis reveals the need for a comprehensive agenda for action to strengthen PHC in EMR. The pandemic commonly triggered new practices and innovations that must be sustained, as well as highlighted gaps in the existing response requiring capacities, resources and better integration. Positive practices catalysed and accelerated by the COVID-19 response include advancing politically championed multisectoral coordination, mainstreaming of digital health innovations (telemedicine and electronic risk communication), expanding cross referrals and disease surveillance across laboratories, hospitals and communities. However, almost 50 years after Alma-Ata, the lack of resourcing and political priority for the PHC approach remains a particular challenge and is most evident in the lack of investment and prioritisation of PC services and community engagement. Countries able to mobilise PC networks as a part of the response provided expanded and quicker access. Notably, higher income countries in EMR have been able to better implement a PHC approach and strengthen PC services, despite it being a cost-effective and best-value way for countries to deliver services.^28^ A distinct feature of these countries is leadership prioritisation of PHC as a whole-of-government and whole-of-society approach to health, as distinct from the discrete service-centric PC. Supported by adequate resources, Group 1 countries demonstrated commitment to strengthen the three interrelated PHC components: integrated health services, multisectoral policy and action and engaged communities.^28^ Findings must be carried forward and linked with health security and UHC reforms commencing now in many EMR countries. Three practice areas for advancing PHC in EMR countries to achieve the goals of UHC and health security are identified in the synthesis.

First, establishing comprehensive PHC-oriented models of care is a critical priority. Findings demonstrate awareness across EMR countries of the centrality of the PHC approach for protecting against disease outbreaks and achieving UHC whereby governments need to prioritise and work towards a comprehensive strategy institutionalising PHC and disease preparedness and response into national strategies.^32 33^ This requires strengthening PC workforces and services standardisation and optimisation, epidemiological support as well as regulation and coordination across levels of care and with the private sector.^3234^,^36^ Global health development partners, including global health initiatives (GHIs), bilateral development agencies and multilateral organisations, can accelerate their commitments to reinforce disease preparedness and response as well as institutionalise PHC within health security. Countries may also need support in managing multisectoral partnerships across the private sector and harnessing the support of civil society.^4 33 34 37^ Additionally, global health partners can also help improve the coordination of aid to reinforce a PHC approach within disease preparedness and response as well as provide support to strengthen legal frameworks and accountability mechanisms.^29 35 38 39^

Second, providing better resources for PC emerges as another priority. Findings showed that EMR countries continue to disproportionately focus on secondary and tertiary care and neglect PC, contributing to disruptions in essential health services during the pandemic and an inadequate PHC response.^33^ EMR countries lag behind in achieving the UHC commitments and public spending on health is only 2.5% of the gross domestic product (GDP) in the EMR, compared with global estimates of 3.5%, further exacerbating disruptions to essential services at the time of health emergencies.^40^,^42^ While several countries (mainly Group 1 and some Group 2 countries, such as Egypt and the I.R. Iran) report progress in improving access to vulnerable populations, the predominance of disease-oriented and hospital-centric financing models continues to deepen health inequities in the EMR and globally.^43^ An emergency funding pool could improve pandemic and disease outbreak response, along with better accountability of health spending to ensure spending on under-resourced areas of the response.^44^ Public financial monitoring systems are commonly limited across countries to track health security spending, have not been digitised and are further constrained by separate siloed pools of GHIs and multilateral support.

Third, strengthening community engagement is a critical component of integrated community-oriented PC. At the same time, there needs to be further efforts to enable community engagement in disease preparedness and response. Involving communities in the design of interventions and establishing bidirectional communication channels and regular feedback mechanisms that demonstrate how input from communities is used in decision-making are essential for meaningful engagement and trust building.^45^ The lack of policy prioritisation to community engagement, poor outreach infrastructure, frontline health workforce shortages and weak involvement of civil society are likely to hamper scale up of community engagement. Digital technology and private sector engagement are important innovations that emerged during COVID-19 and must be sustained for future community engagement.^4 39^

### Implications for research and policy

Findings can help inform national reform efforts and the WHO Regional Office for the Eastern Mediterranean (EMRO) agenda on building resilient health systems to advance UHC and health security, prioritising PHC-oriented models of care supported by enhanced financial protection. WHO EMRO can make progress on its aim to support countries by engaging in capacity building and cross-country lesson sharing and mobilising resources for incorporating findings in national health strategies and disease preparedness and response plans.^33^

Country findings also indicate critical areas of the response that can be strengthened with further and more focused evidence gathering as the basis of a future research agenda to strengthen PHC-oriented disease preparedness and response systems. Telemedicine and digital risk communication emerged as important novel innovations, but less is known about uptake by health workforce and patients, as well as features particularly feasible and acceptable in the local context of EMR countries. The pandemic also highlighted new needs, such as mental health, and further evidence is required on what capacity can be built at the PC tier and how as a part of disease preparedness efforts. Civil society participation and community volunteerism have been traditionally less developed in EMR and new entry points for engagement as well as new social networks of influence that emerged from the pandemic experience must be identified. Multistakeholder coordination, which was a hallmark of the COVID-19 response, is worth unpacking in future studies in terms of key stakeholder motivations, engagement platforms, power-sharing arrangements, legislative and systems support for effective implementation of PHC, in contrast to the discrete service-centric PC.^43 46^ Despite increasing emphasis in the literature on the importance of multisectoral approaches in addressing social determinants of health and promoting equity,^28 43 47^ significant knowledge gaps remain on effective implementation approaches to multisectoral coordination.^43 47^

Finally, further research is needed to understand whether specific response components introduced in EMR countries during the pandemic, including leveraging telemedicine, integrating public health functions with PHC and upscaling multisectoral coordination and community engagement, have been sustained postpandemic. There is a need for a better understanding of how the three PHC components are interlinked and their impact on equity outcomes. Implementing PHC as a comprehensive approach remains highly complex. Gaining insight into which implementation approaches are effective in different settings to enhance equity outcomes can guide PHC implementation efforts, ensuring that they are tailored to local contexts and institutionalised to respond to health emergencies.^43 46^

### Strengths and limitations

This synthesis combines learnings and understanding from case studies to make the most of the data to produce potentially transferable findings to other EMR countries and to other similar settings, including LMICs. A key strength of this synthesis is its findings on the three main components of PHC and the role of PHC in responding to the COVID-19 pandemic. The article also presents a comprehensive agenda, highlighting three priority areas for action for strengthening PHC in EMR.

A limitation of this synthesis is its reliance on the existing case studies that may have varied in the amount of data and the level of detail on context and the role of PHC in the pandemic response, which may limit the findings and their generalisability. To overcome this limitation, individual case studies underwent peer review prior to publication to ensure sufficient methodological information on how data were sourced, collected and analysed. Additionally, all case studies used the three components of PHC framed by the Astana Declaration to guide data collection and analysis and they all provided a background section on the political and socioeconomic factors relating to the unique setting in which the role of PHC was examined, as well as reflected on lessons learnt for strengthening the role of PHC. This has facilitated a coherent synthesis on the role of PHC in EMR countries. We also acknowledge the potential role of researcher bias in the synthesis; however, we aimed to mitigate this through regular discussion of data analysis and interpretation among the researchers (FE-J, NA and AE). Additionally, the authorship team consists of researchers with diverse backgrounds, which helped bring multiple perspectives to the discussion of findings and minimised potential bias.

## Conclusion

Findings revealed notable variations across countries in the extent of PHC involvement in the pandemic response. There is a need for a comprehensive agenda for action and research in EMR countries to institutionalise PHC-oriented disease preparedness and response supported by adequate financing and integrated community-oriented models while sustaining key innovations in digital technology and private sector engagement. Advancing this agenda is critical for achieving UHC and health security, particularly at a time when the EMR is facing various hazards, including conflicts and humanitarian crises.

## Supplementary material

10.1136/bmjgh-2024-017700 online supplemental file 1

## Data Availability

No data are available.

## References

[1] WHO EMRO (2020). COVID-19 situation update February 2020. https://applications.emro.who.int/docs/WHOEMCSR327E-eng.pdf?ua=1.

[2] WHO EMRO (2023). COVID-19 situation report February 2023. https://www.emro.who.int/images/stories/coronavirus/covid_sitrep_feb_2023.pdf?ua=1.

[3] Hammerich A, Fouad H, Elrayah EE (2022). The impact of the COVID-19 pandemic on service delivery for noncommunicable diseases in the Eastern Mediterranean Region. East Mediterr Health J.

[4] El-Jardali F, Fadlallah R, Daher N (2024). Multi-sectoral collaborations in selected countries of the Eastern Mediterranean region: assessment, enablers and missed opportunities from the COVID-19 pandemic response. *Health Res Policy Sys*.

[5] OECD (2021). Strengthening the frontline: how primary health care helps health systems adapt during the COVID-19 pandemic. https://read.oecd-ilibrary.org/view/?ref=1060_1060243-snyxeld1ii&title=Strengthening-the-frontline-How-primary-health-care-helps-health-systems-adapt-during-the-COVID-19-pandemic.

[6] WHO WPRO (2020). Role of primary care in the COVID-19 response. https://apps.who.int/iris/handle/10665/331921.

[7] WHO, UNICEF (2020). Operational framework for primary health care: transforming vision into action. https://www.who.int/publications/i/item/9789240017832.

[8] Alliance for Health Policy and Systems Research (AHPSR) (2025). Primary health care case studies in the context of the COVID-19 pandemic. https://ahpsr.who.int/what-we-do/thematic-areas-of-focus/primary-health-care/primary-health-care-case-studies-in-the-context-of-the-covid-19-pandemic.

[9] WHO (2019). Declaration of astana: global conference on primary health care: Astana, Kazakhstan, 25 and 26 October 2018. https://apps.who.int/iris/handle/10665/328123.

[10] Hardoon D, South J, Southby K (2021). A guide to synthesising case studies. https://whatworkswellbeing.org/wp-content/uploads/2021/04/Guide-to-synthesising-case-studies-2021-FINAL-1.pdf.

[11] Patton MQ (2002). Qualitative research and evaluation methods.

[12] South J, Woodall J, Stansfield J (2024). A qualitative synthesis of practice-based learning from case studies on COVID community champion programmes in England, UK. BMC Public Health.

[13] Dashash NA (2023). Saudi Arabia: a primary health care case study in the context of the COVID-19 pandemic. https://iris.who.int/bitstream/handle/10665/376145/9789240084735-eng.pdf?sequence=1.

[14] Alothman GA (2023). Kuwait: a primary health care case study in the context of the COVID-19 pandemic. https://iris.who.int/bitstream/handle/10665/372729/9789240076686-eng.pdf?sequence=1.

[15] Al Lamki S, Aal Jumaa I, Al Ghafri M (2024). Oman: a primary health care case study in the context of the COVID-19 pandemic. https://iris.who.int/bitstream/handle/10665/376136/9789240088603-eng.pdf?sequence=1.

[16] Syed MA, Al Mujalli H, Ali Abdulmalik M (2023). Qatar: a primary health care case study in the context of the COVID-19 pandemic. https://iris.who.int/bitstream/handle/10665/372729/9789240076686-eng.pdf?sequence=1.

[17] Fikri AM, Suhail A, Al Awadhi T (2022). United Arab Emirates: a primary health care case study in the context of the COVID-19 pandemic. https://iris.who.int/bitstream/handle/10665/366315/9789240064157-eng.pdf?sequence.

[18] El Rabbat M, Hassany M, Abdel-Razek W (2022). Egypt: a primary health care case study in the context of the COVID-19 pandemic. https://ahpsr.who.int/what-we-do/thematic-areas-of-focus/primary-health-care/primary-health-care-case-studies-in-the-context-of-the-covid-19-pandemic.

[19] Takian A, Bakhtiari A, Tabrizi JS (2022). The Islamic Republic of Iran: a primary health care case study in the context of the COVID-19 pandemic. https://iris.who.int/bitstream/handle/10665/366316/9789240063761-eng.pdf?sequence=1.

[20] Zangana G (2022). Iraq: a primary health care case study in the context of the COVID-19 pandemic. https://iris.who.int/bitstream/handle/10665/366290/9789240063785-eng.pdf?sequence=1.

[21] Saadeh R (2023). Jordan: a primary health care case study in the context of the COVID-19 pandemic. https://iris.who.int/bitstream/handle/10665/372727/9789240072930-eng.pdf?sequence=1.

[22] El-Jardali F, Fadlallah R, Shaya R (2022). Lebanon: a primary health care case study in the context of the COVID-19 pandemic. https://iris.who.int/bitstream/handle/10665/366313/9789240058774-eng.pdf?sequence=1.

[23] Berraho M, El Harch I, Benmaamar S (2023). Morocco: a primary health care case study in the context of the COVID-19 pandemic. https://iris.who.int/bitstream/handle/10665/372698/9789240072954-eng.pdf?sequence=1.

[24] Ben Abdelaziz A, Zanina Y, Zoghlami C (2023). Tunisia: a primary health care case study in the context of the COVID-19 pandemic. https://iris.who.int/bitstream/handle/10665/376134/9789240072978-eng.pdf?sequence=1.

[25] Salehi AS, Rahimi AO (2022). Afghanistan: a primary health care case study in the context of the COVID-19 pandemic. https://iris.who.int/bitstream/handle/10665/366289/9789240063747-eng.pdf?sequence=1.

[26] Zaidi S, Hussain SS (2022). Pakistan: a primary health care case study in the context of the COVID-19 pandemic. https://iris.who.int/bitstream/handle/10665/366306/9789240058736-eng.pdf?sequence=1.

[27] Abdel Rahim IM (2023). Sudan: a primary health care case study in the context of the COVID-19 pandemic. https://iris.who.int/bitstream/handle/10665/372734/9789240079595-eng.pdf?sequence=1.

[28] WHO, UNICEF (2018). A vision for primary health care in the 21st century: towards UHC and the sustainable development goals. https://apps.who.int/iris/bitstream/handle/10665/328065/WHO-HIS-SDS-2018.15-eng.pdf.

[29] Rasanathan K, Evans TG (2020). Primary health care, the Declaration of Astana and COVID-19. Bull World Health Organ.

[30] Global Health Security Index (GHSI) (2019). Building collective action and accountability. https://ghsindex.org/wp-content/uploads/2019/10/2019-Global-Health-Security-Index.pdf.

[31] Gale NK, Heath G, Cameron E (2013). Using the framework method for the analysis of qualitative data in multi-disciplinary health research. BMC Med Res Methodol.

[32] van Weel C, Alnasir F, Farahat T (2018). Primary healthcare policy implementation in the Eastern Mediterranean region: Experiences of six countries. Eur J Gen Pract.

[33] WHO EMRO (2022). Building resilient health systems to advance universal health coverage and ensure health security in the Eastern Mediterranean Region. Regional committee for the Eastern Mediterranean 69th session. Provisional agenda item 3(a). EM/RC69/4. https://applications.emro.who.int/docs/Build-resilient-health-systems-UHC-EMR-eng.pdf.

[34] Clarke D, Doerr S, Hunter M (2019). The private sector and universal health coverage. Bull World Health Organ.

[35] Kraef C, Juma P, Kallestrup P (2020). The COVID-19 Pandemic and Non-communicable Diseases-A Wake-up Call for Primary Health Care System Strengthening in Sub-Saharan Africa. J Prim Care Community Health.

[36] Peiris D, Sharma M, Praveen D (2021). Strengthening primary health care in the COVID-19 era: a review of best practices to inform health system responses in low- and middle-income countries. WHO South East Asia J Public Health.

[37] WHO EMRO (2019). WHO’s strategy for the Eastern Mediterranean Region, 2020–2023: turning vision 2023 into action. https://applications.emro.who.int/docs/EMRPUB-RDO-014-2019-EN.pdf.

[38] Witter S, Palmer N, James R (2023). Reimagining the future of global health initiatives. https://d2nhv1us8wflpq.cloudfront.net/prod/uploads/2023/09/FGHI_final_report_designed.pdf.

[39] Kraef C, Kallestrup P (2019). After the Astana declaration: is comprehensive primary health care set for success this time?. BMJ Glob Health.

[40] WHO, IBRD (2021). Tracking universal health coverage: 2021 global monitoring report. https://www.who.int/publications/i/item/9789240040618.

[41] WHO EMRO (2019). Strengthening health financing systems in the Eastern Mediterranean region towards universal health coverage. Health financing atlas 2018. https://iris.who.int/bitstream/handle/10665/311328/EMROPUB_2019_EN_22347.pdf?sequence=1&isAllowed=y.

[42] Jabbour M, Hemadi N, Abou Samra C (2020). Universal health coverage in times of COVID-19 pandemic: actions for emr countries. Knowledge to policy (K2P) center, Beirut, Lebanon. https://www.aub.edu.lb/k2p/Documents/Universal%20Health%20Coverage%20in%20Times%20of%20COVID-19%20Pandemic%20Actions%20for%20EMR%20Countries%20(reduced).pdf.

[43] Edelman A, Marten R, Montenegro H (2021). Modified scoping review of the enablers and barriers to implementing primary health care in the COVID-19 context. Health Policy Plan.

[44] Jain V (2020). Financing global health emergency response: outbreaks, not agencies. *J Public Health Pol*.

[45] Gilmore B, Ndejjo R, Tchetchia A (2020). Community engagement for COVID-19 prevention and control: a rapid evidence synthesis. BMJ Glob Health.

[46] Zaidi S, Zaidi R, Hussain S (2025). Stewarding COVID-19 health systems response in Pakistan: what more can be done for a primary health care approach to future pandemics?. BMJ Glob Health.

[47] Ortenzi F, Marten R, Valentine NB (2022). Whole of government and whole of society approaches: call for further research to improve population health and health equity. BMJ Glob Health.

