# Supplementary material for: Role of a primary healthcare approach in COVID-19 pandemic response in the eastern Mediterranean region: multicountry case study synthesis
Source: BMJ Glob Health. 2025 May 30;10(Suppl 2):e017700. doi: 10.1136/bmjgh-2024-017700 (PMC12128400; doi:10.1136/bmjgh-2024-017700)
Supplement: online supplemental file 1 [file bmjgh-10-Suppl_2-s001.pdf]

Supplementary file 1. Synthesis findings by country

PHC components/ subcomponents	Country classification	Group 1				
	Country	KSA	Kuwait	Oman	Qatar	UAE
How primary care and essential public health functions are responding to COVID-19	Primary care preparedness for COVID-19	(-) PHC workforce and infrastructure were unprepared for emergency, with low pre-pandemic budget allocation (less than 8% of the healthcare budget).<br>(+) Strong leadership support and prioritization of PHC in Models of Care (MOC) pre-pandemic and during pandemic response, with simplified command structures and direct financial access aiding response.<br>(^) Increase PHC budgets to align with MOC goals, enhance PHC workforce training, infrastructure, and emergency preparedness.	(+) Strong political commitment to PHC, comprehensive service packages pre-pandemic, and pre-existing emergency plans (including specific to PHCCs) enabled effective multi-sectoral collaboration and PHC pandemic response.	(-) PHCCs were not fully prepared for emergency , with staff shortages, medication delays, and structural limitations in handling COVID-19 surges. (+) PHC was involved in pandemic preparedness, facilitated by a pre-existing emergency plan, an expanded scope of PHC services pre-pandemic (including management of NCDs and public health functions such prevention and health promotion), and unified government leadership.<br>(^) Prioritize investments to enhance PHC workforce capacity, infrastructure , and emergency preparedness.	(+) Strong political commitment and leadership support to prioritize primary care and multi-sectoral collaboration to improve health outcomes pre-pandemic and a pre-existing national health security plan, including the PHCC Emergency and Disaster Plan, enabled a well-coordinated multi-sectoral COVID-19 response.	(-) Prior to COVID-19, there was no national PHC-specific strategy. A targeted strategy to enhance PHC capabilities and manage surges was developed by the MOH early on in pandemic. (+) Well-established pre-pandemic primary care services, public-private partnerships, and reduced bureaucratic barriers pre-pandemic enabled continuity of care and workforce capacity building during COVID-19.
	Policymaking structures leading the COVID-19 response	• Saudi Arabia's disaster risk reduction system acted at three levels: Strategic (led by the Council of Ministers), Operational (managed by the MOH with financial support from the Saudi Monetary Authority and regional Governorates enabling prompt implementation), and Tactical (involving multisectoral collaboration between government and private sectors).	(+) The pandemic response was centrally governed, with the Prime Minister and Cabinet assigning the MOH to lead response, while other sectors enforced measures. The establishment of a Central Covid-19 Team (CCT), composed of the MOH and all sectors, enabled a central response while decentralizing executive functions. This facilitated evidence-based decision-making, integrated feedback from healthcare facilities, and ensured multisectoral collaboration.<br>(^) Institutionalize this multi-sectoral approach- centralized governance and decentralized executive power- for broader topics such as healthcare services fragmentation.	• A newly constituted Supreme Committee for COVID-19, a multi-sectoral committee chaired by the Minister of Interior Affairs with key participation from primarily the MOH and other relevant sectors, while PHC directors assessed capacity and readiness to guide Committee action. (+) Oman's unified governmental leadership was key to respond to emergency situations.	(+) COVID-19 response was led by the Minister of Public Health through two main structures: the National Multi-ministerial COVID-19 Committee and the Health Strategic Command Group. The Minister of Public Health, assigned the PHC Managing Director to lead the tactical leadership team for health services in the community, which consisted of a number of key partners, and was responsible for planning and managing isolation facilities as well as for ensuring the continuity and sustainability of essential services.<br>(+) The government allocated substantial funding to support the pandemic response, strengthening the overall health system, including primary care.	(+) The COVID-19 Command and Control Center (CCC), responsible for coordinate emergency response with relevant national stakeholders, was led by the MOHAP including PHC in preparedness and response early on.<br>(+) A centralized unified governance and funding approach, dissolved bureaucracies between different departments (e.g., between the Health Information System (HIS), Human Resources (HR), PHC, Finance and Purchasing), allowed for transparent communication channels across all sectors, strengthened PHC response, and swift emergency response.<br>(^) Incorporate task shifting- across sectors- as an early response strategy to enhance flexibility in response and developing a national PHC-specific strategy to ensure a comprehensive and standardized approach to service delivery, optimizing preparedness and resilience for future emergencies.
	Scaling up and managing services related to COVID-19	• PHC played a pivotal role in the COVID-19 response: serving in health quarantines and vaccination centers, modified PHCCs acted as COVID-19 walk-in centers, providing triage, medication, and necessary equipment for suspected cases, while COVID-19 vaccines were made available in public and private PHCCs and pharmacies.<br>(^) Improve public- private partnerships and sustain collaboration for efficient referrals post-pandemic	• PHC and public health played a key role in pandemic response: they were responsible for quarantine and isolation procedures, testing, surveillance, contact tracing, follow- up, public education, and vaccination campaigns and management of COVID-19 cases in designated clinics, with triage units established in every facility to direct patients appropriately and referral policies updated based on feedback from facilities; while also maintaining essential PC services.<br>(+) The existing healthcare infrastructure and homecare/mobile units, including the broad distribution of PHCCs facilitated efficient referral and service load management, and reach to high-risk populations.	• PHC played a key role in COVID-19 response, including: managing COVID-19 cases and referral pathways in coordination with tertiary care, supplying staff to tertiary care, and managing vaccination campaign, with structural changes, a triage system, updated referral pathways, and implementation of the innovative “Tarassud platform” for surveillance, contact tracing, and geofencing, ensuring appropriate service distribution and workload management during the pandemic surge.	• Four PHCCs were designated as COVID-19 health centers: providing screening, testing (through drive-through, mobile testing), and quarantine, while PHCC home health teams maintained physical visits and provided phone consultations, medication delivery. PHCC, in collaboration with other stakeholders, contributed to drive-thru vaccination stations.	• PHC facilities served as the first point of contact for suspected or confirmed COVID-19 cases, with designated centers offering surveillance, screening, COVID-19 testing, contact tracing, managing COVID-19 cases, triage for flu-like symptoms, and managing COVID-19 vaccination, while teleconsultation services and media outreach informed the public of these services.
	Maintaining essential primary care services	(-) Some PHCCs were closed during lockdowns, with staff reallocated to quarantine centers, healthcare workers shortages and high absenteeism contributed to a decline in service efficiency, delays in children's immunization and chronic disease management during the pandemic.<br>(+) PHC workers continued to provide basic services virtually, through the Sehaty app, and medication delivery from PHCCs to patient homes ensured continuity of care. [The pandemic] was an opportunity to re-launch virtual clinics.	(+) The MOH prioritized maintaining essential medical services across all levels of care, especially for NCDs. Walk-in clinics were replaced by telemedicine consultations, with in-person visits continuing when necessary after virtual assessment.	(-) PHC faced workforce shortages due to reallocation to surveillance centers and hospitals, which led to the implementation of “merged health centers”, in which two or more local health centers worked together to provide essential services. There were reports of a decrease in number of visitors to PHCs.<br>(+) Despite disruptions to certain PHC services, essential health services for vulnerable populations, women, and children were maintained. Telemedicine services were initiated at PHCC to sustain access to patients, especially with chronic diseases.<br>(^) Strengthen evidence on telemedicine effectiveness and sustainability and strengthen digital health infrastructure, expand insurance coverage for vulnerable groups, and invest in risk communication and public health expertise for future preparedness and response.	(+) Essential services, particularly for maternal and child health services were maintained, and digital health technology including telephone and video consultations, were implemented to ensure continuity of care.	(+) Essential health services were sustained through drive-through services, telemedicine, home visits, and targeted allocation of “clean” PHC centers for maternal care. The pandemic accelerated the adoption of technology-driven healthcare solutions.<br>(^) Integrate telemedicine into routine care beyond the pandemic, leveraging its cost-effectiveness and accessibility. Explore how AI and digital health tools could enhance emergency preparedness, healthcare efficiency, and financial resilience in volatile markets.
	Essential public health functions (EPHFs)	• PHC staff served in COVID-19 vaccination centers. • Digital platforms served as the main channel for community engagement to improve access to healthcare.	• PHC and PH were engaged early on and were considered instrumental in successfully containing the pandemic through quarantine and isolation procedures, surveillance and contact tracing, public education, and vaccination.<br>• Community volunteers supported PHC response, including: organizing the triage area and patient flow within PHCCs, implementing the electronic appointment system in collaboration with PHC staff, and in vaccination sites volunteers supported data recording, organizing visitor flow and providing instructions to vaccine recipients, as well as helped distribute educational materials and public information.	• PHC workforce was involved in surveillance, managing and tracing cases, vaccination, with community engagement in health promotion and education.<br>(-) Weaknesses in response included lack of experts in risk communication and community engagement, insufficient public health cadre, and underdeveloped digital platform for disease surveillance and insurance coverage for vulnerable populations.	(+) Enhanced PHC infrastructure for surveillance and contact tracing, through advanced Information Technology (IT) solutions that collected real time demographic information, symptoms and contact history of individuals who presented for COVID-19 PCR tests, and was integrated with other existing databases.<br>• Volunteers were deployed to implement public health measures in the communities, such as wearing of masks and maintain social distancing in supermarkets. • PHCC raised awareness of COVID-19 precautionary measures, vaccination, and encouraged continuity of care and contributed to drive-thru vaccination stations, in collaboration with other stakeholders.	• PHC teams rapidly developed and implemented strategic plans to maintain public health functions and PHC facilities were allocated for surveillance, screening, and contact tracing, and vaccination; while teleconsultation services and media outreach informed the public of these services.<br>(+) Public trust strengthened and empowered the integration of public health within PHC services.<br>(^) Ambulatory care services could be integrated to cover public health and PHC activities, demanding better use of health workforce, improved development and training, effective budget allocation and enhanced facilities to deliver services efficiently.
How multisectoral policy and actions were involved in responding to COVID-19	Role of primary care in multisectoral programs/initiatives	• Multisectoral coordination across governmental organizations, including the Council of Ministers, the MoH, the Monetary Authority, regional level authority, and with private-sector entities.<br>(+) Engagement of the private sector by providing support to the healthcare enabled an effective pandemic response.	• The Central Covid-19 Team (CCT), a governance body composed of a spectrum of MOH leaders and experts from all sectors including Public Health (PH), Primary Health Care (PHC) and Preventive Medicine (PM), played a crucial role in coordinating multisectoral collaboration, developing evidence-based policies, ensure the engagement of all stakeholders at multisectoral and intersectoral levels, mobilizing the nations' resources within the public and private sectors, and adapting response to the evolving pandemic.<br>(+) Multi-sectoral action was facilitated ported by: leadership from the Prime Minister and Cabinet; direct communication between governance bodies and PHC facilities; and the pre-existing national plan for emergency preparedness and response that actively engaged PHC services, ensuring a holistic approach to addressing the health needs of communities and outlining clear roles, responsibilities, lines of accountability and chain of command to guide PHC and other sector response to support vulnerable populations and disseminate accurate information.	• Multi-sectoral coordination through an emergency multi-sectoral committee was crucial to manage simultaneous response to the cyclone and pandemic. Collaboration between PHC and other public sectors supported PHC vaccination efforts. Collaboration with the private sector provided in expanding PHC capacity for testing and vaccination through providing healthcare workforce. Multi-sectoral collaborative response was effective in reducing community transmission, reducing number of infections, hospital admissions, and deaths.<br>(+) Unified governmental leadership, high political commitment, policy transformation, and early community engagement in response to the pandemic enabled an effective response.<br>(-) There is a need to improve health literacy and communication and information sharing between policymakers and the community.<br>(^) Sustain the progress achieved in multi-sectoral collaboration, high political commitment, policy transformation, and early community engagement to build a comprehensive One Health approach.	• The National Committee for Coronavirus (NCC), chaired by the Minister of Public Health with membership reflecting a cross-government approach, ensure that all sectors provided support to the health sector, maintaining essential and critical services, engaging the public and raising their awareness, and trust in government. Cooperation across government agencies played a role in employee vaccination, relieving burden from PHCC and enabling continuity of essential services.<br>(+) A pre-existing National Health Strategy (NHS) 2018-2022 emphasizing multi-sectoral cooperation, pre-existing health cities programs, and a flexible multisectoral collaborative governance structure, with a hub-and-spoke model (with the hub being the NCC and the spokes being the sector leads) and a top-down and bottom-up approach, supported by strong leadership enabled a coordinated response.	• Two official bodies (NCEMA and CCC), included representatives from all relevant entities to enable direct and cross-channel communication, and were tasked with coordinating the national effort across various sectors.<br>(+) Pre-existing strategic financial partnerships, multisectoral policies, standard operating procedures (SOPs), and memoranda of understanding (MOUs), between the public and private sectors supported the supply of a workforce to address rising demand for health services in PHCCs.<br>(+) Partnership with the MOHAP leading the CCC was critical for the effective implementation of COVID-19 response and recovery action plans.<br>(+) Transparent communication channels between government levels and NGOs ensured coordinated action, while the Unified Financial System (UFS) enabled efficient resource allocation.
	Collaborations involving donors/development partners					
To what extent engaging and communicating with communities to leverage community resources was effective	Role of primary care in empowering communities	• Community volunteers were recruited through a MOH volunteer platform, received infection control training, and supported the MOH response by organizing admission to quarantines, health education, risk communication, screening support, data collection, and reporting. Volunteers received basic infection control training.<br>(+) Actively engaging the population in pandemic response enabled an effective pandemic response	• (Pre-existing) Homecare services, within the PHC setting, provided care to vulnerable populations, with electronic records of these individuals, was utilized reach high-risk populations through PHC mobile units.<br>• Community-based volunteers, were recruited through existing platforms and trained to provide support to PHC staff in vaccination sites (data recording, organizing flow, providing instructions and distribution of MOH educational materials and public information, medication delivery).<br>• The pre-existing Healthy City Program (HCP) facilitated rapid response by leveraging community engagement to support PHC response. Volunteers organized triage area and patient flow within the PHCC and implement the electronic appointment system in collaboration with PHC staff, and supported establishment of a field hospital.<br>(+) Strong pre-pandemic volunteer networks enabled and pre-established community partnerships [and healthcare facilities] within Healthy Cities enabled effective preparedness and response	• Civil societies and associations were strong partners to PHC throughout the pandemic though providing medical equipment for vulnerable populations to continue their treatment at home, and volunteers supported vaccination. Pre-existing Healthy Cities, introduced by the MOH in collaboration with community- based groups for the implementation of public health interventions, engaged the community to support PHC response in disseminating information to the public, enhancing public communication, awareness and compliance with national health policies.	• Community- based volunteers, recruited through a volunteering campaign, provided support in mental health, logistics and raising awareness, and ensuring implementation of public health measures.<br>• Pre-existing well- established structures to engage with the local communities, for example, PHCCs has ‘health centre friends’ as community advocates representing communities. These structures were used to map communities and identify vulnerable groups in the context of COVID-19 in order to engage them and inform surveillance, testing, and vaccination policies.	• The PHC role in terms of community engagement utilized the media and digital platforms to reach all communities and conduct surveys and assessments. Community engagement through gauging public response to COVID-19 measures and using the information for reviewing guidelines and measures. Public engagement with the PHC system has increased since the peak of the COVID-19 pandemic because awareness has been raised on the services provided.<br>(+) Transparency and frequent communication with the public to ensure access for socially vulnerable populations, active engagement of communities and other stakeholders, and implementation of the COVID-19 response.<br>(+) Simplified, purpose-built governance mechanisms and protocols [was key to effective response].
	Community engagement approaches/mechanisms	• Digital platforms served as the main channel for community engagement.	• Volunteer participation was facilitated through established online platforms<br>(+) Open and consistent communication channels were used, including official websites, social media, hotlines, and public announcements, were used to disseminate information and foster community confidence. Well-established bidirectional communication channels (as above) and feedback loops, such as surveys, public forums, and helplines, enabled the government to understand community needs and concerns.	(+) PHC engaged the community through Pre-existing healthy cities (HCs) and Healthy Villages (HVs). (^) There is a need for strengthened communication between policymakers and communities.	• Volunteering campaign and well-established structures within PHCC to engage with the community (health centre friends). • PHCC's communications team provided unified messages to the public on accessing healthcare services and COVID-19 through social media, SMS etc.<br>(-) Ehteraz application helped monitor compliance with pandemic measures. However, different channels of communicating messages to the diverse population may be necessary.	• Communication channels exist between the government and the community. For example, the government developed long-standing strategies for communication with the community, including open-door policies and national surveys and assessments. Telecommunication and media platforms (including social media) were made accessible and integrated with public health services to reach the whole of the community.<br>(+) The existing telecommunication infrastructure enabled optimal use for different services and the dissemination of risk communication throughout the pandemic.<br>(^) Open communication channels between key actors and entities are likely to be critical to ease challenges as they arise during future health crises.

PHC components/ subcomponents	Country classification	Group 2			
	Country	Egypt	I.R of Iran	Iraq	Jordan
How primary care and essential public health functions are responding to COVID-19	Primary care preparedness for COVID-19	(+) Strong political leadership, alongside UNICEF's collaboration with MoHP, facilitated the development and the implementation of the preparedness and response strategy on all fronts, including training, equipment and vaccine procurement, and community outreach.	(-) Political and economic factors (including sanctions), preference of policymakers for secondary- level care, inadequate staff training, equipment, personal protective equipment (PPE), and coordination among groups limited the development of the PHC sector. (+) Strong laboratory systems, surveillance data accessibility, and emergency preparedness and response planning prepandemic contributed to facilitating pandemic response.<br>(^) Improve capacity for implementing response plans, risk communication, and financing.	(-) Political instability and financial factors, absence of crisis preparedness planning, inadequate PPE and medical supplies and their geographical distribution, disproportionate focus on hospital workers for support and training for emergency preparedness, and weak integration between national and subnational governance for PHC planning (For example, the subnational committee comprised solely of politicians who worked independently from national structures), as well as the historical marginalization of PHC and prioritization of hospital and specialist care hindered effective response.	(-) Poor political will, limited funding, inadequate capacity for emergency response, and reliance on NGOs to implement PHC strategies negatively impacted service continuity and PHC effectiveness in the pandemic. (+) Past historical commitment to PHC, as key to UHC and service provision to refugees, and collaboration between USAID and MOH to enhance COVID-19 response through expanded testing, infection prevention and control measures, and training for healthcare workforce facilitated the engagement of PHC in emergency response.<br>(^) Optimize PHC governance and leadership and adequate PHC funding to enhance pandemic response
	Policymaking structures leading the COVID-19 response	• The COVID-19 response was led by the Office of the President through a the Higher Committee for the Management of the COVID-19 Pandemic (headed by the Prime Minister) and the High-Level Oversight Committee (led by the Minister of Health), which was responsible for planning, multi-sectoral collaboration, and implementation of response.	• The COVID-19 response was coordinated through the COVID-19 National Committee (CNC), headed by the President with representatives from relevant ministries, while COVID-19 Provincial Committees were set up for localized response.	• Covid-19 response was initially led by the Minister of Health through a Crisis Cell, which was later replaced by the Higher Committee for Health and National Safety, headed by the Prime Minister, including several ministers. I4	• The National Epidemic Control Committee (NECC), which brought together experts from the MoH, academia, and the Royal Medical Services to oversee developments and provide recommendations to decision-makers.
	Scaling up and managing services related to COVID-19	• The response strategy shifted focus over time from hospital-centric care to prioritizing the role of PHC in COVID-19 response to increase surge capacity and maintaining essential services, through: introducing new models of care, screening, contact tracing, surveillance, education and vaccination, and managing COVID-19 cases (through telephone and domestic visits), facilitated by reorganization of flow pathways and staff schedules, segregating care pathways, and task shifting of PHC staff to isolation sites, leveraging telemedicine, and engaging in outreach activities through virtual channels, mobile unites, domestic visits, medication home delivery to ensure care for vulnerable populations.<br>(^) Establish an integrated information system linking all levels of care with social determinants of health to support decision-making, strengthen political support and investments in PHC, including financing reforms, optimize service distribution across urban and rural areas, and evaluate successful pandemic initiatives and models of service delivery adopted by the PHC system, for scale up and building resilience to future emergencies.	• Specific PHC centers were designated as COVID-19 centers, providing screening (also through mobile units), contact tracing, outpatient care, and vaccination, thus reducing hospital overcrowding and strengthening the resilience of the healthcare system; while other PHCCs provided routine care.<br>• The Health Observatory Center was established within PHC centres to aggregate and analyse data.<br>(-) Delays in fully utilizing PHC capacity in pandemic response and the voluntary referral system posed challenges as patients bypassed the gatekeeping process, directly accessing higher levels of care.	(-) The hospital-centered approach to pandemic response and pre-existing weaknesses in the PHC system, including limited access to essential health services, limited capacity to swiftly scale up emergency response. Inadequate testing capacity and lack of formal referral pathways contributed to late patient presentation and high fatality rates.<br>(+) The establishment of mobile medical teams to provide COVID-19 care, replacing PHCCs and difficult to access hospitals.<br>(^) Develop a robust health information system (HIS) supported by a national policy or strategy for e-health and information, communication and technology (ICT).	• The government, with WHO and EU support, strengthened PHC to maintain essential health services and support COVID-19 response, targeting the vulnerable populations through providing vaccinations, diagnostic tests for COVID-19, referrals of COVID-19 suspected cases, surveillance of COVID-19.<br>(-) The lack of a well-defined PHC delivery model and staff shortages impacted the continuity of care.<br>(^) Sustain PHC strengthening initiatives, establish care quality monitoring teams to oversee patient flow, and accelerate the implementation of the national telemedicine program to enhance remote healthcare access and service continuity.
	Maintaining essential primary care services	(+) While hospital and critical care were initially prioritized, over time, efforts were made to increase surge capacity and mobilize PHC to maintain essential services, facilitated by strong community trust in CHWs. Digital tools and databases improved service delivery and medication delivery. Investments were made to enhance PHC facilities, and targeted outreach efforts ensured care for vulnerable populations.<br>(^) Expand and institutionalize innovations like digitized services (e.g. telemedicine) and home visits to strengthen PHC.	(-) Interruption of routine health care services, particularly for NCD.<br>(+) The PHC structure was redesigned to provide access to vital health services (separate access to COVID-19 and routine service centres), with primary care packages delivered either in-person or remotely. Mental health and nutritional consultants delivered PHC services and provided targeted assistance to vulnerable households.<br>(+) The Integrated Health Portal (SIB)- the PHC network's health information system- and the application of telehealth services in response to COVID-19 as well as the Syndromic Surveillance System (SSS) provided the information needed to plan public and specific evidence-based interventions and enabled identification of vulnerable groups and provision of targeted access to essential services, despite data quality issues.<br>(^) Strengthen integration of health information systems across PHC, secondary care, and private health sectors and improve the telemedicine infrastructure to provide cost-effective, scalable healthcare beyond the pandemic and ensure service continuity during future emergencies.	(-) Closure of PHCCs and cessation of primary care services, due to lockdown measures and a historical focus on hospital-based care over PHC, with disruptions to maternal and child care and mental health and with 65% of older adults struggling to access medication.<br>(+) Some PHC teams provided virtually via online platforms and telephone calls, helping to mitigate the impact of lockdowns.<br>(+) Integrate community-led initiatives- such as information gathering, basic service provision and awareness-raising activities into PHC.	(-) Lockdown measures at the initial phase of the pandemic resulted in disruption of essential services, mainly due to gaps in emergency preparedness and limited training for healthcare workers.<br>(+) PHC centers quickly rearranged services and implemented infection control measures, allowing them to resume essential care, including vaccinations and prenatal care. The Ministry of Health (MoH) prioritized maintaining routine PHC programs and community health services, ensuring continued access to maternal and child health, mental health, and emergency care.<br>(^) Mobilize financing and enhance budget allocation to support the integration of PHC models particularly for vulnerable populations, integrating digital health technologies for service delivery, and sexual and reproductive health, and mental health.
	Essential public health functions (EPHFs)	• PHC teams supported contact tracing, surveillance and vaccination and conducted health education campaigns with communities and vulnerable groups.<br>(+) Trust between CHWs and their communities and digital applications facilitated dissemination of health instructions; while linkages and referrals across different levels of care, supported the integration of primary care and public health functions.	• PHC was involved in testing and screening, utilizing the SIB information system was used for screening and follow up, vaccinations, and conduct surveys to inform public health measures.<br>• PHC network recruited and trained volunteers to support contact tracing, screening, community education, and COVID-19 vaccinations.<br>• Digital health technology, such as Mask App and web-based screening, were utilized for health prevention and screening.	• Volunteer mobile medical teams were established to visit families and patients affected by COVID-19 to provide consultations and treatment including oxygen cylinders.	(-) Lack of computer systems and electronic connectivity for crisis management at the national level hindered surveillance and contact tracing, partly due to lack of funding and low–level of priority provided to IT infrastructure.<br>(^) There is a need for an effective and advanced information system to identify, detect and communicate the spread of infections, health demands, and shortage in service.
How multisectoral policy and actions were involved in responding to COVID-19	Role of primary care in multisectoral programs/initiatives	• Multisectoral policies and actions were initiated with the support of the Higher Committee, headed by the prime minister, and endorsed by the involved ministers and stakeholders at national and subnational levels.<br>• Multisectoral action involving fiscal and social policies were implemented to protect the vulnerable population and ameliorate the socio-economic impacts of the pandemic. For example, collaboration between the National Council of Women (NCW) and government partners supported the implementation of mitigation and response policies to ensure the protection of women and girls.<br>• The COVID-19 pandemic provided an opportunity to emphasize the role of PHC in strengthening health systems and in community and multisectoral responses to health emergencies.	• Multisectoral collaboration and the creation of PHC operational and community teams (including CTTs, HCTs, and monitoring and support teams) were key during the pandemic, addressing SDH, through several social and fiscal policies: PHC facilities connected vulnerable populations with organizations for providing food and financial aid, government financial assistance and tax cuts. Private sector providers providing human resources for 24-hour centres and routine services to target groups.<br>(-) Poor coordination mechanisms, insufficient communication to inform the public and civil society about PHC priorities and unclear roles and priorities weakened multisectoral collaboration.<br>(^) Establish incentives to encourage partners to participate in multisectoral collaboration, document the multisectoral experiences and innovations of the pandemic for future action plans, establish the Alliance of PHC Friends, comprising people, organizations and NGOs interested in supporting PHC to reach vulnerable people, strengthen the PHC structure and incorporate integrated and lifelong services.<br>(^) Comprehensive plans could address SDH and ensure practical multisectoral collaboration to revitalize the PHC network as the engine for health system strengthening to achieve UHC.	• New national, subnational and local structures were created to facilitate communication, coordination, and planning for COVID-19 response.<br>(^) Institutionalize multisectoral action to promote health across all policy frameworks.<br>(-) Arguably, the failure to engage the PHC system in addressing these multisectoral challenges represents a missed opportunity.<br>(-) Barriers were highlighted concerning the integration of public and private sectors into governance and communication processes.	• Multisectoral collaboration in Jordan was quickly adopted in the pandemic and included shared services, information, policies, and efforts.<br>(+) The Primary Health Care Strategic Plan of Jordan for 2018 – 2022 included important actions of multisectoral partnership that is concerned with the management of COVID-19 and the achievement of SDGs. The MoH established a monitoring and evaluation department within the PHC to the evaluate current strategies, plans, and COVID19 response.<br>(^) The crisis management unit of the MoH integrated a strategic preparedness plan including the participation of all stakeholders for emergency response [but this was established after COVID-19].<br>(^) Collaborative action should be part of national plans and clearly informed to all sectors with clear responsibilities<br>(^) Integrate PHC delivery model that uses the capacity of the system efficiently through integration of multi-sectoral action with the private sector, NGOs, and community stakeholders to provide continuous care and respond to emergencies. This integration should occur at the organization level through coordinated provider networks overseen by the MoH.
	Collaborations involving donors/development partners	• International organizations, UN agencies, and NGOs streamlined their responses to ensure harmonization with the national-level strategy. The pandemic triggered a whole-of-government approach to boost national production of supplies, medicines, and vaccines, in coordination with international bodies, including GAVI and COVAX.<br>• UNICEF in collaboration with MoHP supported the COVID-19 response and COVAX vaccination through procurement of vaccine and hygiene materials, workforce training and community outreach activities to increase awareness and services accessibility and support the refugee community.<br>(-) Weak linkages between the MoHP and NGOs and delayed inclusion of some stakeholders, such as private sector, challenged multisectoral pandemic response.<br>(^) Enhance role of the non-government sector in coordinated partnerships with the PHC system and strengthen mechanisms to promote social inclusion, decrease disparities and reduce impact on vulnerable groups.	• Iran approached the international Humanitarian Buffer mechanism established within the COVAX facility to provide 1.6 million doses of COVID-19 vaccines for Afghan refugees. Donors and charity organizations were crucial in providing PPE and equipment to special centres.		• PHC inclusion in emergency response actions was achieved through the collaborative work of the USAID with the MoH to prepare public hospitals, private hospitals and clinics to identify and manage COVID-19 cases, expand infection prevention and control measures, strengthen testing, and implement communication campaigns.
To what extent engaging and communicating with communities to leverage community resources was effective	Role of primary care in empowering communities	• CHWs and mobile teams from PHC facilities formed an integral part of the communication and health education plans for on-site and outreach interventions.<br>• The PHC Facilities Board, including local community representatives, was activated in the governorate of Port Said, where Egypt's new social and health insurance programme was being implemented. This approach enabled the involvement of the community in decision-making, facilitated the identification of local vulnerable groups, and allowed bidirectional communication.<br>• The COVID-19 pandemic has provided an opportunity to emphasize the role of PHC in strengthening health systems and in community and multisectoral responses to health emergencies.	• Community-based PHC volunteers received basic training and helped to educate people, screen and contact tracing, monitor compliance with public health regulations, and support vulnerable groups. Basij forces helped to distribute masks and oxygen supplies at the beginning of the pandemic. Under the MoHME's guidance, Basij promoted home-based screening and supported PHC teams in COVID-19 vaccination, distributed masks and oxygen supplies.<br>• Each home, one health post: Pre- pandemic, the PHC network initiated the Health Ambassadors' project to enrich people's health literacy, improve healthcare access, increase emergency preparedness and strengthen public participation in health systems. The project assigned one member from each household to receive health education from PHC facilities and voluntarily act as an ambassador to educate the rest of the family on health matters. Health ambassadors now cover 50.1% of households, and more than 250000 participated in the COVID-19 response. It played a vital role in keeping the community safe, improving access to services, and strengthening health system resilience.<br>• Contact tracing teams (38% from the health department, 33% Basij, 16% Health Ambassadors and 9% local volunteers), led by the PHCCs, tracked COVID-19 cases, reviewed symptoms, conducted rapid tests, and made referrals.<br>• Home-care teams (74% from the health sector, 19% Basij, and 3.3% private sector) performed tests to people at home, provided medicine and made referrals.<br>• Support teams (78% Basij, 12% Health Ambassadors, 5.6% community health volunteers and 2% Red Crescent Society) assisted vulnerable families, introducing them to medical centres or support organizations, and providing financial and dietary aid.<br>(+) Intersectoral collaboration, training and efforts to strengthen NGOs, social networks and media participation enabled contact tracing in the community and limited virus spread.<br>(+) The National Risk-Communication and Community Engagement Strategy for COVID-19 was designed based on the national flu response programme, with two-way conversation covering essential COVID-19 information and community feedback.	• Civil society organizations (CSOs) played a critical role in channeling community response towards the pandemic. For example, some CSOs suspended their activities to focus on the COVID-19 response, through fundraising to provide food aid and other essential services e, and monitor the government's policies. Volunteer mobile medical teams<br>provided consultations and treatment to patients including oxygen cylinders.<br>(^) Institutionalize CSO participation and community engagement in service provision, policy-making and government structures through legal provisions. Formalize Community-based initiatives – such as information gathering, basic service provision and awareness-raising activities – within the function of PHCCs, for example, community hubs could be established that link to PHCCs and that operate as a form of PHC response to public health emergencies.<br>(-) Public mistrust in the government and widespread corruption weakened community engagement in response.	• Pre-pandemic, weak community engagement led the MoH to establish a ‘community health committee’ to encourages communities to use and participate in healthcare services and advocate for community needs, especially during emergencies. These committees were not leveraged during the pandemic.<br>• IPC/WASH programs implemented by the MOH in PHCCs helped raise awareness of communities on safety measures.<br>• The COVID-19 Youth Volunteers' Taskforce was established as a partnership between the Ministry of Interior, UNDP, and the Government of Norway to encourage youth to engage in the COVID-19 response.<br>• Community-based organizations (CBOs), smaller organizations at the local level, provided education to the community.<br>• The MoH adopted a Stakeholder Engagement Plan (SEP) to support PHC pandemic response.<br>(^) A community-based system connected to public information systems can help prevent information fragmentation. Engage communities in caring for vulnerable populations and reporting back to the MoH through an interactive communication/information system. The MoH started implementing healthy villages program, which aims to empower communities and is planning to include community institutions in disease prevention and surveillance.
	Community engagement approaches/mechanisms	• A package of risk communication and community engagement was developed with the support of NGOs and UN agencies, it prioritized maintaining two-way communication and engaging with trusted CHWs to reach vulnerable populations.<br>• The Egyptian Red Crescent Society, through its 2000 trained volunteers, assessed societal health risks and the infodemic to support vaccination.<br>(+) Communication and community engagement teams worked to increase public trust in the pandemic response, building on pre-existing trust between CHWs and their communities, although variations were perceived in outreach activities across governorates.<br>(^) Integrate community engagement as a central component of a PHC approach to improve accountability, community empowerment, and responsiveness of the PHC system and assess outreach activities and communication channels leveraged during the pandemic (including via clear grievance mechanisms) to inform future emergency response and improve transparency and public trust in the health system.	• Primary channels for COVID-19 risk communication were public health volunteers and various hotlines, administered by the PHC network. Official and public media, official websites (e.g., webda.behdasht.gov.ir), social media and other information, education and communication (IEC) materials were also utilized.<br>(-) Insufficient coordination and integration between groups and sectors and lack in training in risk communication led to conflicting messages and often confused the public.<br>(^) Improve communication channels and skills between healthcare providers (doctors, nurses, etc.) and the community, and between national health managers and both provincial providers and the community.		• Channels of communication with the community included public and private mass communication channels, social media, and religious institutions' preachers. (^) Adapt digital technologies to effectively and feasibly engage stakeholders during crises, building a network of communication and information exchange system with the MoH and other national entities

PHC components/ subcomponents	Country classification				
	Country	Lebanon	Libya	Morocco	Tunisia
How primary care and essential public health functions are responding to COVID-19	Primary care preparedness for COVID-19	(-) Lack of political and economic stability resulting in inadequate PHC funding and marginalization, historic low investments in PHC, disproportionate focus on strengthening hospital capacity, expansion of private healthcare services, inadequate PHC workforce training and equipment, and absence of a national PHC-specific strategy and PHC-tailored guidelines relating to the COVID-19 response contributed to increased risk of community transmission.<br>(+) Although not formally integrated in the COVID-19 response, the PHC Department at the MOH provided training to PHC workforce on COVID-19 and equipped centres with necessary supplies, with support from WHO Lebanon and the Order of Nurses.<br>(^) Develop a national PHC-specific strategy to mobilize PHC centres during emergencies, secure political support to prioritize PHC, and allocate resources to strengthen PHC as the foundation of emergency response and community health.	(-) Shortages of medical supplies and PPE in PHCCs, along with limited guidance and training at the PHC level, hindered effective pandemic response.<br>(+) Support for securing PPE was provided by municipalities and local donors helped to address shortages for the limited available staff.<br>(^) Strengthen preparedness by ensuring clearly defined leadership and roles, availability of strategies, guidelines, and training, and essential supplies for future emergencies.	(-) Limited healthcare financing and shortage of healthcare workers, with disparities in regional distribution, expansion of the private health sector with underdeveloped public-private partnership weakened the pandemic response. (+) Support from international organizations facilitated acquisition of medical equipment for continuity of essential PHC services, workforce training, strengthening PHC structures, and development PHC guidelines and monitoring tools.<br>(^) Invest in expanding and equitably distributing human resources for health while leveraging international support to sustain essential PHC services and improve preparedness for future crises.	(-) Lack of trust in public institutions, shortages in health infrastructure, medical supplies, and outdated PHC capacity, limited to prevention and lacking community participation and intersectoral collaboration, and further strained by the expansion of the private sector and the prioritization of a hospital-centric approach limited pandemic response.
	Policymaking structures leading the COVID-19 response	(-) The MoPH developed a COVID-19 Health Strategic Preparedness and Response Plan, collaborating with relevant authorities and partners, with lack of recognition or explicit role for the PHC Department at the MoPH and the National PHC Network, limiting resources for primary care. (^) Strengthen MoPH stewardship functions to harness existing PHC resources and capacities, coordinate and integrate efforts across the diverse response agencies (public sector, NGOs, private sector), and steer the overall health system response.	(-) The Higher Multi-Sectoral Committee (led by the Minister of Health and supported by the NCDC scientific committee) was initially responsible for overseeing response. However, a parallel Scientific Advisory Committee was later established by the MOH, becoming the main decision-maker. The lack of a unified strategy led to improvised decisions, parallel systems, weak coordination, lack of clarity on the role of the MOH, and PHC decisions were ad hoc, made by local health offices level, without central guidance.<br>• In 2021, with appointment of the Government of National Unity was appointed and the NCDC scientific committee resumed its advisory role to the MoH.	(+) The COVID-19 response and multi-sectoral collaboration were led by the MoH and supported by an expert advisory committees enabling evidence-informed decision-making.<br>(+) The integration of PHC into national preparedness and response plans strengthened linkages and referrals across different levels of care.<br>Centralized management by the MoH enhanced coordination, minimized inefficiencies, and reinforced the government's capacity to lead the crisis response.	(-) A centralized government- led response favoring a hospital-centric response to the pandemic marginalized 40 years of PHC practice.<br>(-) The division of healthcare responsibilities between the MOH (planning) and the Ministry of Social Affairs (funding) contributed to fragmented decision-making, broader factors such as public trust, governance transparency, information systems, and political stability also influenced effectiveness of PHC response.<br>(+) Eventually PHC played a critical role in preventing the collapse of the national healthcare system through inclusive, participatory, and collaborative practices<br>(^) Strengthen and integrate PHC into future emergency preparedness and response, given PHC proximity to communities and the public's trust in PHC.
	Scaling up and managing services related to COVID-19	(-) Beyond awareness-raising and screening of patients, PHC played no major role in the pandemic response, with several challenges: a hospital-centric approach to preparedness and response, absence of clear referral pathways leading to variations in practice across PHCCs, low adherence to screening protocols and incomplete data reporting, and fragmented information systems and poor connectivity hindering real-time data sharing and limiting follow up with patients. PHC was not involved in COVID-19 vaccination. Additional activities were mostly self-initiated depending on existing capacities and resources, exacerbating inequitable healthcare access.<br>• The PHC Department at the MoPH was engaged in the development and dissemination of COVID-19-related messages, select PHCCs engaged in screening and follow- up, and some PHCCs demonstrated innovation in response through leveraging telemedicine and segregating care pathways.<br>(^) Strengthen PHC information systems (link PHC information systems with the national surveillance system), establish a real-time information-sharing platform linking PHCCs, hospitals, and public health organizations, and evaluate COVID-19 response initiatives to embed lessons learned into future emergency preparedness.	• At the start of the pandemic, only 10% maintained normal services, with 5% handling suspected cases and emergencies, with weak referral and coordination across PHC, laboratories, surveillance teams, and other health facilities.<br>• PHC provided detection points for suspected cases, monitoring teams, mobile medical teams for follow up of home cases, swabs' taking points, and referral services (to a limited extent).<br>• COVID-19 vaccination was based in PHC facilities, supported by mobile teams.	• PHC was integrated into pandemic preparedness and response, including COVID-19 screening and diagnosis, contact tracing, referral and follow-up of cases, education, and COVID-19 vaccination, with specific protocols for suspected and confirmed COVID-19 cases established in PHCCs,<br>(-) The involvement of PHC in COVID-19 response reducing [provision of] other services and programmes, particularly in the context of insufficient human resources.	• During the early waves of the pandemic, PHC played a passive role, with many shutting down or acting as “stop-gap” to referral facilities, a focus on a hospital-centric approach to preparedness and response, inadequate referral procedures, and movement restrictions and care seeking-behavior further impacting disease burden. The first contact with PHCCs was for obtaining the fourth booster of COVID-19 vaccine.<br>• PHC workers were redeployed as vaccinators in immunization megacities and new local clinics, or in small mobile teams to provide screening services at homes of elderly people with limited autonomy.<br>(^) Integrate new health programmes, utilizing digital technologies and multidisciplinary teams into PHC models of care.
	Maintaining essential primary care services	(-) Closure of some PHCCs, reduction in care-seeking anxiety, mobility restrictions, and financial constraints contributed to disruption essential service and a decline service utilization. Unavailability of advanced telemedicine options (e.g., videoconferencing or remote patient monitoring), poor internet coverage, electric power shortages, and low digital health literacy further restricted access to remote care. Additionally, disparities in PHC capacity led to inequitable service provision, with remote and underserved areas suffering from staffing and material shortages.<br>(+) However, 86% of PHCCs under the National PHC Network remained operational, providing subsidized PHC consultations to 36% of vulnerable Lebanese, and the Syrian and Palestinian refugees. Additionally, PHCCs engaged in outreach efforts via phone and WhatsApp, following up with chronic disease patients, and modifying medication distribution through extended prescriptions and home delivery services.<br>(^) Integrate PHC services with mental health and social care services (including geriatric care, GBV, youth-friendly services) to enable more comprehensive and integrated care.	(-) At the start of the pandemic, preventive and curative services, including routine childhood immunizations, were severely disrupted due to weak referral guidelines, insufficient training, and PPE shortages. Referrals and home visiting services were inadequate, with poor coordination between the National Center of Disease Control (NCDC), laboratories, surveillance teams, PHC teams, and other health facilities.	(+) Morocco reorganized its health system, integrating PHCCs into national preparedness and response plans, ensuring the continuity of essential services such as vaccination and maternal health, while also using telemedicine to improve accessibility.<br>(-) The increased reliance on PHC during the pandemic [for both COVID-19 response and maintaining essential services] led to reductions in other health services programs, particularly in the context of insufficient human resources.	(-) The closure of PHCCs, PHC staff redeployment, mobility restrictions, drug shortages, and inadequate referral procedures severely disrupted essential services, particularly impacting the continuity of care for chronic conditions and preventive services, during the early stages of the pandemic.
	Essential public health functions (EPHFs)	• The PHC Department at the MoPH was engaged in the development and dissemination of COVID-19-related messages as well as information, education and communication (IEC) materials. Despite the lack of integration of the PHC network in the formal pandemic response, some PHCCs contributed to screening and contact tracing.<br>(-) Absence of unified health care information system and limited national capacity for surveillance and random mass testing at large scale hindered real-time data-informed decisions, additionally underinvestment in the PHC network manifested in the inadequacy of primary care and public health integration efforts.<br>(^) Strengthen the linkages between PHC services and public health and provide additional training to PHC staff, strengthen the surveillance function of primary care, including provision of real-time alert systems for managing infectious diseases and enabling planning and mobilization at the required scale nationally.	• PHC provided detection points for suspected cases and COVID-19 vaccination	• Primary care was responsible for COVID-19 screening and diagnosis, case investigation and contact tracing, reporting of cases, sensitization and education of the population, and the COVID-19 vaccination campaign.	• PHC staff reassigned as vaccinators in immunization megacities, or in mobile teams to provide screening services at homes of vulnerable populations. (^) Epidemic management in PHC: preparedness, response and resilience functions for epidemics/pandemics can be better integrated into PHC facilities across different health regions.
How multisectoral policy and actions were involved in responding to COVID-19	Role of primary care in multisectoral programs/initiatives	• Inter-Ministerial Emergency COVID-19 Response Committee headed by the Prime Minister, coordinated multi-sectoral action. However, there were no representatives from the PHC Network within this Committee and its mandate and working methods are unclear.<br>• The lack of formal integration of the PHC network into municipal-level responses was a missed opportunity for leveraging the resources and capacities of PHCCs.<br>• Structured multisectoral coordination of PHC with public health, municipalities, community-based services and secondary care was lacking in the COVID-19 response.<br>• Some PHCCs collaborated with the education sector to raise awareness within school and community settings. However, these efforts were self-initiated and ad hoc.<br>(-) Challenges hindering the multisectoral response include: fragmented authorities and multiplicity of actors; competing interests and competition for resources; weak formal coordination structures; and limited resources and funding for implementation of response; absence of data automation systems across sectors and actors, and a poor culture of information sharing.<br>(^) Integrate PHCCs as part of multisectoral response units/committees at both national, subnational and local levels with specific roles and responsibilities.	• Local multi-sectoral action, represented by health offices in the municipalities, focused on equipping the PHCCs with needed supplies.<br>• Municipalities formed local emergency committees, including heads of local sectors, local volunteers and experts and led by the Mayor or a municipality counselor. PHC decisions were taken locally by local health offices and municipalities and were ad-hoc, with no central guidance.<br>(+) There was good communication between health offices, municipalities and donors at local level for provision of some essential supplies, but (^) [this coordination at local level needs to be] sustained to strengthen service delivery and community participation.<br>(-) Lack of a unified strategy led to improvised decision, parallel systems, and weak coordination, lack of clarity on the role of the MOH.	• Morocco reorganized its health system and proactively pursued multisectoral collaboration and community engagement to support the response effort.<br>• Multisectoral collaboration was strengthened to address the pandemic's health, social and economic consequences. An emphasis on primary care was included in the national and territorial governance of the crisis, which strengthened linkages and referrals across different levels of care.	• Multisectoral collaboration was well-conducted despite little involvement from PHC/BHC facilities.<br>• Community-led initiatives and multisectoral actions – essential for mobilizing the population during pandemics– were not sufficiently valued.
	Collaborations involving donors/development partners	• The international community (i.e., intergovernmental and humanitarian agencies, notably UN agencies and WHO) played a vital role in the pandemic response, in terms of securing PPE and essential supplies and addressing the social and economic burden of the pandemic, including among vulnerable populations like refugees. This was essential in the absence of governmental social safety nets in a country with a privatized model of social welfare and under financial strain.	• Support of the development partners was suboptimal and patchy [also with respect to PHC]. • Local donors helped provide supplies to PHC.	• Efforts to strengthen PHC during the pandemic were supported financially by international organizations (WHO, UNICEF), the Spanish Agency for International Development Cooperation (AECID) and the Global Fund) within the framework of international cooperation in 2020.<br>• The support focused on the synthesis of strategic priorities resulting from a forum on PHC, a National PHC Strategy, and equipping PHC facilities, as well as evaluating these efforts.<br>(+) Pre-established links with regional and global health authorities and international development and humanitarian agencies was a key factor to secure medical supplies and vaccines and workforce training.	
To what extent engaging and communicating with communities to leverage community resources was effective	Role of primary care in empowering communities	• Some PHCC's established community advisory groups to discuss pertinent community health needs and inform their PHCCs. These were activated to identify vulnerable populations during the pandemic.<br>(-) However, these committees are not always present or active. As such, the integration of community engagement is still lacking as a central component of the PHC approach to strengthen accountability, transparency and responsiveness of the PHC system.<br>• Most PHCCs engaged with communities to disseminate health education and awareness on COVID-19, in coordination with municipalities and volunteers.<br>• While volunteers played an important role in PHC centres' outreach activities, recruiting volunteers was challenging amidst the economic crisis and PPE shortages.<br>(^) Integrate community engagement as a central component of a PHC approach to improve participation, accountability, transparency and responsiveness of the PHC system.<br>(^) Promote better coordination among local and international NGOs, UN agencies and governmental organizations to reduce fragmentation, enhance the flow of resources, and increase accountability, effectiveness and impact of public health response.	• Municipalities called for community-volunteers, they helped with disinfection, supporting families, and providing community awareness.<br>• Localities' chiefs act as the link between the community and PHC.	The majority of PHCCs engaged with their communities (through local government officials, schools, and volunteers) to disseminate health education and awareness messages on COVID-19; volunteer doctors provided remote medical advice; and civil society organizations mobilized their capacities to support the elderly, to combat violence against women and children, and to provide social protection to vulnerable groups.	• Community engagement – which was officially led by the central facilities of epidemiology and directly involved the MoH – was not as successful [as multi-sectoral collaboration].<br>• Community-led initiatives and multisectoral action – essential for mobilizing the population during pandemics – were not sufficiently valued.<br>• Civil society, professional organizations and official agencies were mobilized in support of health personnel, through developing digital applications to support healthcare staff. Civil society organizations were mobilized via social networks to manage the distribution of oxygen condensers to patients at home.
	Community engagement approaches/mechanisms	PHCCs demonstrated innovation in their responses through engaging in outreach activities conducted through several virtual channels.		(+) Communication channels enabled through new technologies allowed integration of community feedback into activities and policies at all levels.	• The government sought to build trust with younger generations through transparent and proactive communication and support.

PHC components/ subcomponents	Country classification	Group 3			
	Country	Afghanistan	Pakistan	Sudan	Yemen
How primary care and essential public health functions are responding to COVID-19	Primary care preparedness for COVID-19	(-) Longstanding shortages in PPE and medical equipment, weak testing capacity and surveillance and vaccine distribution systems, absence emergency preparedness plans for PHC hindered pandemic response.<br>(+) International support significantly contributed to vaccination coverage, securing medical resources, and facility improvements for COVID-19 management.<br>(^) Strengthen PHC workforce and infrastructure to integrate within the broader healthcare delivery system, while strengthening PHC capacity for emergency preparedness and response.	(-) Scant urban PHC infrastructure for managing COVID-19 in densely populated low-income areas, insufficient equipment and training, and weak pandemic response planning structures, and weak health emergency networks [with limited funding] hindered pandemic response.<br>(+) Large network of PHC and secondary facilities supported by community-based health workers and a robust formal private sector facilitated healthcare delivery.<br>(^) Institutionalize emergency preparedness and response by strengthening existing IHR structures, disease surveillance units, and health emergency networks with dedicated leadership, governance, and legislative support, and adequate resources.	(-) Significant inequity in healthcare access between urban and rural areas, healthcare worker shortages, chronic underfunding, political and economic instability, and prolonged neglect, inadequate preparedness, poor training and lack of PPEs and logistical support to workforce led to PHCCs closures.	(-) Limited healthcare facilities, severe workforce and supply shortages, weak governance, corruption, ongoing conflict, political and economic instability, heavy reliance on external funding, and focus on hospitals for case management hindered pandemic preparedness and response and the mobilization of PHC in pandemic response.<br>(+) Support from WHO and international donors helped mitigate challenges by providing training, medical supplies and PPE, field hospitals, and financial allowances for healthcare workers.
	Policymaking structures leading the COVID-19 response	(-) Sporadic funding for the MoPH crisis response unit, decentralized pandemic response to provincial authorities with limited capacity, and delays in engaging NGOs and the private sector weakened pandemic response.<br>(^) Integrate CHWs in emergency preparedness and response.	(-) The COVID-19 response was led at the executive level by non-health stakeholders (through the National Command and Control Centre (NCOC), headed by the Prime Minister), without investment in building dedicated leadership and response centres within the health sector.<br>(+) However, executive-level leadership provided unity of purpose across multiple sectors, additionally, collaborative governance, data-driven decision-making helped to effectively surge capacity, procurement, and response monitoring.<br>(^) Institutionalize capacity for health emergencies within the MoNHSRC and provincial DoH, strengthen competencies, and develop interoperable information systems.	(-) Inter-sector coordination was ensured through the participation of all ministries in the higher Pandemic Containment Committee, while the Emergency Operations Committee at the FMOH led daily decision-making with technical and material support from technical subcommittees and the WHO and UN agencies; however, despite PHC representation in the national committee and given support, its role was largely limited to maintaining essential service delivery.	(+) The Scientific Committee, established by the MOPH&P, developed a case management protocol and referral pathways in collaboration with WHO.
	Scaling up and managing services related to COVID-19	(-) Despite commitment of MOH to maintain essential services and strengthen PHC response, the PHC response faced significant challenges, including lack of on-site testing in most health facilities, shortages of medical professionals, logistical challenges in vaccine distribution, and the inability to conduct effective contact tracing or enforce home-based care and referrals. Additionally, pre-existing community-based networks were not integrated into the surveillance system, which remained limited to urban areas, leaving many cases undetected.<br>• Rapid response teams were deployed for contact tracing within the community.	(-) PHC integration in response was largely overlooked, as the focus was on tertiary hospitals, which weakened screening, referrals, patient counseling, quarantine enforcement, and risk communication at the local level.<br>(+) Areas of relative success of the pandemic response include building up clinical surge capacity and using digital data for decision-making and boosting local production and supply chains. Direct community engagement, largely undertaken by NGOs that had existing networks in place, and mobilizing community-based volunteers and religious leaders for risk communication [during later waves].<br>(^) The COVID-19 pandemic accelerated the adoption of digital technologies for risk communication, telemedicine support and virtual triaging platforms, cell phone tracking systems for contact tracing, with various levels of uptake and success [these can be evaluated and lessons learned used for future scale up]	• Despite PHC representation in national committees, PHC role was limited to maintaining essential services, following a period of abrupt PHCCs suspension, due to hospital- centric preparedness and response, workforce shortages, and movement restrictions. Although the PHC directorate developed guidelines for service continuity and triage, these plans were not immediately implemented due to abrupt service suspensions, though they were later adapted upon resuming care.<br>• Instead, Rapid Response Teams (RRTs)- composed of a doctor, nurse, public health officer and a lab- to respond to calls or information about suspected cases, followed by a site visit for testing and referral; however, the RRTs [were not able to reach adequate response levels].	• While many PHCCs shut down during the pandemic, with 51% of health facilities remaining fully functional, of those 20% provided maternal and child health care and 15% were repurposed for COVID-19 response (e.g., triage rooms) and their role restricted to screening and referrals, while hospitals managed confirmed cases.
	Maintaining essential primary care services	• The COVID-19 pandemic initially disrupted PHC service delivery, particularly affecting care for noncommunicable disease (NCD).	(-) There was disruption to essential services and decline in healthcare utilization, including for maternity care, immunization, and preventive services, due to inadequate PPE and IPC training, health provider and patient hesitancy, as well as lack of a concerted preparedness strategy to ensure uninterrupted delivery of PHC services in an emergency.<br>(+) The Forum for Motherhood – a coalition of health practitioners, UN agencies and development partners working in reproductive, maternal, newborn and child health- played a key role in maintaining RMNCH services, supporting health workers with PPE, training, and SOPs. Telemedicine and digital media use grew, especially for mental health, aiding consultations and risk communication.<br>(-) The sustainability of the multisector response for mental health remains highly questionable.	(-) Health services were suspended during the early phases on the pandemic, PHC's role was restricted to maintaining essential services, while hospitals received the majority of resources. The use of digital technology and telemedicine to provide essential services was limited in scope and many patients could not be reached for logistics or unfamiliarity reasons.<br>(+) Select PHCCs were refurbished, equipped, and re-opened, and provided with transport and financial incentives for staff, this was followed home delivery of medication and telemedicine consultations for MCH and chronic disease patients.<br>(^) The crisis highlighted the importance of PHC services and the potential of digital health solutions for future development of PHC services.	(-) PHC services were halted at the peak of COVID-19, with only 20% of health facilities providing integrated maternal and child health care, leading to a decline in consultations, surgical procedures, and child vaccinations due to PPE shortages and fear of infection.
	Essential public health functions (EPHFs)	• Some PHC health facilities provided vaccination services, but faced with staff shortage and logistic issues.<br>(^) Incorporate case management in PHCCs as well as other public health measures including disease notification and surveillance, and preventative actions including vaccination, and provide an integrated crisis response package enabling emergency preparedness and response.	(+) A network of designated public and private laboratories was established for rapid reporting of cases and digital data systems provided rapid and comprehensive data analytics, including statistics on COVID-19 testing for evidence-based planning. • Direct community engagement, largely undertaken by NGOs that had existing networks in place, and mobilizing community-based volunteers and religious leaders for risk communication [during later waves].<br>• The COVID-19 pandemic accelerated the adoption of digital technologies for risk communication, telemedicine support and virtual triaging platforms, cell phone tracking systems for contact tracing, with various levels of uptake and success .<br>(^) Integrating essential public health functions such as surveillance, health promotion, community engagement, and emergency preparedness and response is challenging and needs to be informed by country insights on opportunities and barriers.	(^) Integrate public health functions and emergency response with PHC, as the most convenient approach to reach the entire population, quick coordination with national level, and a cost-effective emergency response.<br>(^) PHC staff needs to be offered appropriate training to adequately undertake the new functions while the PHC facilities need to be provided with necessary resources and tools to undertake new mandates.	• The role of PHC facilities was limited to screening and referral.
How multisectoral policy and actions were involved in responding to COVID-19	Role of primary care in multisectoral programs/initiatives	(-) Despite establishment of a committee to manage the COVID-19 crisis, Afghanistan was unsuccessful in its attempts to coordinate the pandemic response. The absence of collaboration among different sectors led to disruption and fragmentation within the national response, with delays in action and a general lack of public trust.<br>(-) Policy decisions pertaining to the participation of nongovernmental organizations (NGOs) and the private sector were delayed.<br>(^) Institutionalize multisectoral action, through policies, and review mechanisms for whole-of-government engagement to maintain essential health services during emergencies and to tackle wider determinants of health. This could include coordination at the national and provincial levels and between sectorial ministries, and in partnership with NGOs and humanitarian actors.	• The COVID-19 response was mobilized quickly and delivered using a multisectoral approach within a heavily decentralized context.<br>(+) Enabling features of the multisector response included the overarching leadership and legitimacy provided by the National Command and Control Centre (NCOC) and its chairs, the Minister of Planning and Development and Provincial Chief Executives; the health sector provided critical technical advice, data and guidelines to shape the multisector response; decentralized funding across sectors enabled an agile response; and data sharing and visual data analytics helped with procurement and local supply.<br>(+) High-level leadership and political championing of the NCOC led to a quick pandemic response based on joined-up action across multiple sectors as well as national– provincial coordination.<br>• Areas of relative success include public–private engagement. Service delivery was enhanced by government stewardship of private health providers to meet surge inpatient and testing capacity. This involved a mix of new private-provider engagement mechanisms from strategic coordination for data sharing and cross-referrals for ambulance support, to the designation of private laboratories as testing centres and the procurement of hospital-based services.	• Multisectoral collaboration for the pandemic was coordinated through the national (ministerial) coordination committee, representing all ministries. • The collaboration between the ministry of finance and the MOH was described as unprecedented in terms of its generosity.<br>The private sector mobilized support which helped physical rehabilitation of facilities.	• The Higher Committee was established to coordinate the pandemic multi-sectoral response, with members from different sectors. Local authorities also coordinated multisectoral activities in their locality.<br>(-) The multi-sectoral [action] was considered weak because the Committee is a [temporary] structure for crisis management and not a sustainable mechanism.<br>(^) There is a need of a national preparedness and response plan and a formal body to respond to health emergencies.
	Collaborations involving donors/development partners	(+) Help from the international community proved to be a critical factor in enhancing vaccination coverage in Afghanistan	• Private mobile networks have partnered with UN agencies, the government and NGOs for public messaging on COVID-19 issues, with several million users engaging via Facebook, Twitter and the digital apps.<br>• The Forum for Motherhood – a coalition of health practitioners, UN agencies and development partners working in reproductive, maternal, newborn and child health (RMNCH) – was instrumental in the protected opening up of public and private RMNCH services and in the provision of PPE, training and SoPs support to RMNCH staff.<br>• A multisector, UN-supported WASH cluster is in place and the National WASH strategy and existing resources were re-aligned throughout 2020 and 2021 as part of the COVID-19 response.<br>(-) [Multi-sectoral efforts with development partners] were not guided by a deliberative strategy, however, meaning that hunger mitigation efforts between government, philanthropic and development partners remained largely uncoordinated during the period under review.	• International assistance from WHO, UNICEF, MSF etc. complemented community efforts enabling strengthening the cold chain capacity, supporting to online services and mass media communication, and provision of greatly needed equipment, supplies.	• Different humanitarian “clusters” have been established over time to coordinate efforts of UN agencies, and national and international NGOs. The National Health Cluster was formalized in Yemen in 2010 to meet population health needs and coordination of humanitarian response, but its focus shifted to respond to the pandemic.<br>• Humanitarian and development partners, coordinated by UN OCHA, supported disease surveillance, deployed dedicated COVID-19 staff within agencies, tracked the impact on routine essential healthcare, developed messaging to encourage behavioural change, and boosted ICU capacity.
To what extent engaging and communicating with communities to leverage community resources was effective	Role of primary care in empowering communities	• The government and the PHC system in Afghanistan failed to engage the pre-established community healthcare workers (CHWs) system (more than 25 000 CHWs throughout the country, providing a link between the community and the health care system, responsible for health promotion, delivery of specific health services and referrals).<br>(^) Communities could be better engaged as a standard practice within the PHC system in emergencies response, requiring the development of suitable and reliable systems prior to, during and after a crisis.	• Suspension of community-based programmes and outreach by community health workers contributed to decreased access to care<br>• Direct community engagement was undertaken by NGOs that had existing networks in place. For the COVID-19 response, NGO activities were coordinated through a health cluster set up by UN agencies. NGOs were either subcontracted by UN agencies to provide community engagement activities through their established networks or organizations undertook self-funded philanthropic activities.<br>• Considerable community volunteerism was seen during the initial response to COVID-19 to provide food, social care and health services to the growing low-income populations. Youth groups were perceived as community change agents during the pandemic response, with their digital literacy.<br>(^) NGOs, CSOs and outreach health programmes provided important resources for community preparedness, local government stakeholders need to be involved more systematically in community response activities.<br>(-) Engagement was ad hoc at times, lacked common messaging for risk communication and service referrals, was insufficiently resourced to sustain support, and lacked planning for integration with PHC service delivery. Local government was a particularly weak link in community engagement that needs to be strengthened systematically.	• Despite well-established traditions of community organizations and participation in PHC delivery and health promotion and prevention, these were not fully mobilized during the pandemic.<br>(-) However, the apprehension associated with COVID-19 was inconducive to mobilize community engagement, the centralized top down approach required less input from the community, and the abrupt suspension of health services, particularly PHC centers, as the attention of funding was shifted to hospital/ emergency response limited opportunities of engaging with the community.<br>• Yet, the community mobilized resources from the private sector and Sudanese diaspora to equip hospitals. Community- based volunteers provided support to the elderly and vulnerable populations.<br>(^) Incorporate community level engagement within the PHC mandate, requiring training of PHC staff and adequate equipping of facilities.	• Community engagement, at the PHC level, takes different forms, including community outreach, volunteerism and NGO participation, as well as informal youth initiatives (e.g. young volunteers distributed oxygen cylinders to patients at home during the pandemic), and community health volunteers supporting nutrition. However, as a consequence of COVID-19, some engagement activities stopped, while NGO activities shifted from a focus on routine services to COVID-19 emergency response.<br>• Since 2006 to 2020 the outreach approach supported provision of basic health services for children under 5 years in the most vulnerable communities, responding to financial risk protection challenges and enhancing efficiency in the provision of health services. However, outreach activities stopped at the peak of the pandemic.<br>• Donor's support to all PHC activities were reduced, due to the funding being shifted to the COVID-19 response.<br>• health sector organizations encouraged NGOs to work with communities, an informal youth volunteering initiative distributed oxygen cylinders to patients at home during the pandemic.
	Community engagement approaches/mechanisms	(-) The public mistrusted authorities due to a lack of governmental competency in providing timely information about COVID-19, the spread of misinformation and rumours, and previous erroneous and unsatisfactory pledges by the government.	• Risk communication and community engagement (RCCE) in public health emergencies establishes dialogue between the health authorities and at- risk populations for risk management. In Pakistan, an RCCE Taskforce for COVID-19 was set up by the MoNHSRC with membership from UN partners and technology partners to establish a single technical source of scientific communication and guidelines. However, most community engagement occurs in the provinces and there remains a disconnect between the national RCCE effort and community engagement on the ground.<br>• Diverse modes of communication were used but with a strong shift towards digital platforms for the general public and face-to-face engagement by NGOs in targeted communities. Mass media and social media were used extensively by the public sector, national celebrities, medical associations and political parties during the three COVID-19 waves to give targeted messages, while radio announcements, mosque announcements, banners and pamphlets were used by NGOs in underserved communities.<br>• Innovative solutions such as mobilizing community-based volunteers from the polio network, engaging religious leaders for incorporating COVID-19 risks and preventative measures into religious sermons and youth groups for peer-to-peer awareness were also used to promote public awareness of COVID-19, alongside helplines to inform callers about testing and tracing cases and to obtain feedback.		

(-) Barriers, challenges
(+) Facilitators
(^) Lessons learned or recommendations
